# Optimizing in-situ upgrading of heavy crude oil via catalytic aquathermolysis using a novel graphene oxide-copper zinc ferrite nanocomposite as a catalyst

**DOI:** 10.1038/s41598-024-73953-0

**Published:** 2024-10-28

**Authors:** Ahmed Ashraf Soliman, Mostafa E. Aboul-Fetouh, Sayed Gomaa, Tarek M. Aboul-Fotouh, Attia Mahmoud Attia

**Affiliations:** 1 Mining and Petroleum Engineering Department, Faculty of Engineering, Al-Azhar University, Nasr City, Cairo, 11884 Egypt; 2 Petroleum Engineering Department, Faculty of Engineering and Technology, Future University in Egypt, Cairo, 11835 Egypt; 3 Chemical Engineering Department, Faculty of Engineering, British University in Egypt (BUE), El Sherouk City, Cairo, Egypt; 4 Petroleum Engineering and Gas Technology Department, Faculty of Energy and Environmental Engineering, British University in Egypt (BUE), El Sherouk City, Cairo, Egypt

**Keywords:** Heavy crude oil, In-situ upgrading, Catalytic aquathermolysis, Graphene oxide-based nanocomposite, Enhanced heavy oil recovery, Fossil fuels, Catalysis

## Abstract

Unconventional resources, such as heavy oil, are increasingly being explored and exploited due to the declining availability of conventional petroleum resources. Heavy crude oil poses challenges in production, transportation, and refining, due to its high viscosity, low API gravity, and elevated sulfur and metal content. Improving the quality of heavy oil can be achieved through the application of steam injection, which lowers the oil’s viscosity and enhances its flow. However, steam injection alone falls short of meeting the growing demand for higher-quality petroleum products. Catalytic upgrading is therefore being investigated as a viable solution to improve heavy oil quality. This study experimentally investigates the application of two novel catalysts, namely copper-substituted zinc ferrite (ZCFO) synthesized via the sol–gel combustion method and a graphene oxide-based nanocomposite (GO-ZCFO) with different ratios, for catalyzing aquathermolysis reactions in the steam injection process, with the aim of enhancing the in-situ upgrading of heavy oil. These catalysts underwent characterization using X-ray powder diffraction (XRD), Fourier Transform Infrared Spectroscopy (FTIR), and Transmission Electron Microscopy (TEM). Their catalytic performance was assessed utilizing a high-pressure/high-temperature reactor (300 ml), with a comprehensive analysis of the changes in the physical and chemical properties of the heavy oil before and after upgrading. This analysis included measurements of sulfur content, SARA fractions, viscosity, API gravity, and Gas Chromatography (GC) of saturated hydrocarbons and evolved gases. All upgrading experiments, including both catalytic and non-catalytic aquathermolysis processes, were conducted under a reaction time of 6 h, a reaction temperature of 320 °C, and high pressure (86–112 bar). The results indicated that the introduction of the proposed catalysts as additives into the upgrading system resulted in a significant reduction in sulfur content. This, in turn, led to a decrease in resin and asphaltene content, an increase in the content of saturated hydrocarbon, particularly low-molecular-weight alkanes, and ultimately, a reduction in viscosity along with higher API gravity of the crude oil. GO-ZCFO with a weight ratio (50:50) exhibited the best catalytic performance. The heavy crude oil, upgraded with this 50:50 ratio, exhibited significant enhancements, including a 29.26% reduction in sulfur content, a 21.27% decrease in resin content, a 37.60% decrease in asphaltene content, a 46.92% increase in saturated hydrocarbon content, a 66.48% reduction in viscosity, and a 25.49% increase in API gravity. In comparison, the oil upgraded through non-catalytic aquathermolysis showed only marginal improvements, with slight reductions in sulfur content by 5.41%, resin content by 3.60%, asphaltene content by 11.36%, viscosity by 17.89%, and inconsiderable increases in saturated hydrocarbon content by 9.9% and API gravity by 3.02%. The GO-ZCFO, with its high catalytic activity, stands as a promising catalyst that contributes to improving the in-situ upgrading and thermal conversion of heavy crude oil.

## Introduction

As conventional oil resources diminish and global energy demands increase, unconventional oil sources are emerging as a promising solution to fulfil the rising need for petroleum products. Projections indicate that conventional oil production will be insufficient to meet the world’s growing energy requirements, underscoring the critical role of unconventional oil in addressing future energy challenges^1^.

Heavy oil, an unconventional hydrocarbon resource with an API gravity lower than 20°, has become a key energy source for the twenty-first century. Substantial reserves of heavy oil represent a noteworthy portion of unconventional resources, alongside other sources such as coalbed methane, tight gas, shale gas, and hydrates^2^. Several countries, including Canada, Venezuela, Russia, the United States, and China, have significant reserves of heavy oil^3,4^. The largest accumulations of heavy oil on a global scale are found in Canada and Venezuela. Western Canada holds approximately two and a half trillion barrels, Venezuela has around one and a half trillion barrels, Russia possesses roughly one trillion barrels, the US holds between one hundred to one hundred eighty billion barrels, and China has around seventy-three billion barrels^5^.

Over the past decade, heavy oil production has significantly increased due to market demands and advancements in drilling and recovery technologies. As a result, the efficient extraction of heavy oil has gained greater importance. However, the challenging aspect of heavy oil recovery lies in its high viscosity^6,7^. Currently, the production of heavy crude oil from reservoirs through the primary recovery method is limited to approximately 3–10% of the initial oil in place (IOIP)^8^.

Besides, the utilization of waterflooding as a secondary recovery method can only recover around 5–10% of the initial oil in place (IOIP). This limited recovery is due to a substantial portion of heavy oil being bypassed during waterflooding, which is primarily caused by challenges such as water fingering and coning. These issues arise from the un-favourable mobility ratio between the displacing fluid (water) and the displaced fluid (high-viscosity oil)^9^.

Owing to the drawbacks associated with traditional oil recovery methods in terms of low oil production, Enhanced Oil Recovery (EOR) techniques are increasingly being employed as an alternative approach to augment the amount of oil extractable from oil reservoirs^10–12^. These techniques include chemical flooding (polymer, surfactant, alkaline or combinations)^13–23^, gas flooding (carbon dioxide)^24–27^, and thermal injection (steam or in-situ combustion)^28,29^. In the pursuit of more effective and economical approaches to recovering heavy oil, the oil and gas industry has focused on advanced technologies, particularly thermal recovery methods. Thermal recovery encompasses the application of heat and pressure to the heavy crude oil within the reservoir, causing it to be converted into lighter and more valuable crude oil. This process not only increases the amount of extractable oil but also enhances its quality^30,31^.

Nowadays, the steam stimulation process is the most successful and commonly applied thermal recovery technique for the in-situ upgrading of heavy oil reservoirs. Steam injection involves introducing steam into the reservoir to heat the heavy oil, reducing its viscosity and enhancing its flow to the production well^32^. Research has shown that steam injection results in both physical and chemical effects. From a physical standpoint, steam injection employs well-established drive mechanisms, including high pressure, high temperature, and viscosity reduction. Chemically, the process involves a series of chemical reactions that take place among heavy crude oil, minerals in the reservoir rock, and high-temperature water or steam. These reactions, known as aquathermolysis, break down long-chain hydrocarbons into smaller ones, thus ultimately lowering both the viscosity and molecular weight of heavy crude oil. The key mechanism of aquathermolysis is the cleavage of C–S bonds at high temperatures, facilitated by their low bond energy, leading to a decrease in viscosity^33–35^.

During steam injection for enhanced heavy oil recovery, several mechanisms can cause heat losses, which affect process efficiency and ultimately reduce the amount of recoverable oil. Consequently, the extent of aquathermolysis reactions becomes very limited^33^. To mitigate these heat losses, catalysts can be employed to accelerate chemical reactions by reducing the activation energy required. This results in increased cracking of specific types of bonds, such as C–S, C = S, C–C, C = C, C–N, C = N, C–O, and C = O, present in heavy oil^36^. Hence, the reactions between heavy crude oil, reservoir rock minerals, and steam, facilitated by catalysts, can be referred to as “catalytic aquathermolysis”^37,38^.

## Literature review

Several experimental studies have been carried out with the aim of enhancing the process of in-situ upgrading of heavy crude oil. These studies have focused on exploring the feasibility of using nano-sized metals as catalysts to upgrade heavy oil in situ. The use of nanoparticles in this process is pivotal in advancing the efficiency and sustainability of hydrocarbon recovery. Nanoparticles, with dimensions ranging from 1 to 100 nm and a surface area to volume ratio up to 1000 times larger than that of microparticles, possess unique chemical properties such as high adsorption affinity, improved catalytic activity, dispersibility, and intrinsic reactivity^39^. Additionally, the non-porous surface of nanoparticles reduces the degree of deactivation and poisoning, thereby enhancing their catalytic performance and increasing the probability of contact^40,41^.

In a study conducted by Hamedi Shokrlu et al. (2010)^42^, the use of nano-sized metals (nickel, iron, and copper) to reduce heavy oil viscosity during thermal applications was investigated. They found that metal nanoparticles could significantly decrease viscosity even at room temperature, with a more pronounced effect during steam stimulation due to enhanced catalytic activity in aquathermolysis reactions. Later, Hamedi Shokrlu et al. (2013)^43^compared the catalytic effects of nanometer-sized nickel and micron-sized Raney nickel on viscosity reduction during steam injection applications. Both catalysts effectively facilitated the aquathermolysis process, enabling in-situ upgrading. However, nickel nanoparticles outperformed Raney nickel due to their higher surface-to-volume ratio, which results from their smaller size. In their study, Al-Marshed et al. (2015)^44^ optimized heavy oil upgrading using iron oxide (Fe_2_O_3_) nanoparticles. The study found that under the conditions of 42 °C, 50 bar, 60 min, and 0.1 wt.% catalyst, the iron oxide catalyst significantly enhanced the conversion of heavy crude oil into lighter, more valuable products. Omajali et al. (2017)^45^ studied the impact of bio-nanoparticles derived from gram-positive and gram-negative bacteria on heavy oil upgrading. They found that Pd and Pd/Pt bio-nanoparticles outperformed non-metallized biomass, non-catalytic thermal processes, and commercial Ni-Mo/Al_2_O_3_nanoparticles by reducing coke formation and increasing liquid yields. Although both types of bacteria showed similar catalytic performance, bio-nanoparticles from gram-positive bacteria were preferred for large-scale production due to their ease of synthesis. Lam-Maldonado et al. (2020)^46^evaluated the catalytic activity of transition metal nanocatalysts, including Ni, Mo, and Fe nanoparticles, synthesized using a modified inverse microemulsion method for heavy oil upgrading. The synthesized nanocatalysts enhanced the API gravity of the heavy oil from 13° to 18°, facilitated asphaltene conversion rates between 20 and 43%, and achieved moderate removal of sulfur and nitrogen. Suwaid et al. (2020)^47^ investigated the effects of oil-soluble transition metal catalysts (Fe, Co, Ni) on heavy oil upgrading during steam injection. Autoclave experiments showed that these catalysts transformed into metal-based complexes, such as oxides and sulfides, at 250 °C and 300 °C, thereby enhancing aquathermolysis reactions. At 300 °C, the catalysts, particularly nickel-based ones, significantly reduced the viscosity from 2034 to 1031 mPa^.^s, increased the saturated hydrocarbon content, and decreased the levels of resins, asphaltenes, sulfur, nitrogen, and polyaromatics. AL‑Rubaye et al. (2022)^48^ evaluated bimetallic catalysts (CuFe_2_O_4_, CoFe_2_O_4_, and NiFe_2_O_4_) for steam-based enhanced oil recovery. They found that NiFe_2_O_4_was particularly effective at 300 °C, reducing viscosity, increasing saturated hydrocarbon content, enhancing the H/C ratio, and facilitating both desulfurization and denitrogenation. Suwaid et al. (2022)^49^examined oil-soluble copper-based catalysts with various organic ligands (oleate, stearate, decanoate, and octanoate) for upgrading heavy crude oil. Copper oleate, in particular, demonstrated optimal performance at 300 °C, resulting in a 50.31% reduction in viscosity, an increase in saturated hydrocarbon content from 29.4% to 41.12%, and significant reductions in resin and asphaltene content. Wang et al. (2022) and Li et al. (2022) both explored the effects of catalytic modification on heavy oil upgrading. Wang et al. (2022)^50^ developed cis-9-octadecenylamine-modified α-Fe_2_O_3_ and Fe(OH)_3_nanoparticles, demonstrating that these catalysts were effective in reducing the viscosity of Shengli extra heavy crude oil. Meanwhile, Li et al. (2022)^51^ synthesized Fe_3_O_4_ particles via co-precipitation and modified them with 3-propyl trimethoxysilane (KH570), oleic acid (OA), and triethoxyvinylsilane (A151), resulting in Fe_3_O_4_–KH570, Fe_3_O_4_–OA, and Fe_3_O_4_–A151. Their study showed that these modifications achieved viscosity reductions of 27.91% for Fe_3_O_4_–KH570, 51.27% for Fe_3_O_4_–OA, and 29.44% for Fe_3_O_4_–A151 in Chenping heavy oil. Al-Muntaser et al. (2022)^52^investigated the role of decalin as a hydrogen-donor solvent in the catalytic and non-catalytic hydrothermal upgrading of heavy oil. The experiments were conducted at 300 °C and 1044 psi over a 24-h period. The results indicated that using decalin in conjunction with nickel (II) stearate as a catalyst led to a substantial 69.2% reduction in viscosity, a decrease in the content of resin and asphaltene, an enhancement of low-molecular-weight alkanes, and the removal of sulfur and nitrogen. Mikhailova et al. (2023)^53^ studied the catalytic potential of Ferrocene-based ligand (Mono-, Di-, and Tri-Ferrocene) in the hydrothermal upgrading of Tatarstan heavy crude oil at 300 °C and 72 bar. Tri-Ferrocene achieved a 40% reduction in viscosity and improved the hydrogen-to-carbon ratio, while Mono-Ferrocene transformed into Fe_3_O_4_(magnetite) and FeS, which acted as active catalytic phases in the hydrothermal conversion. Liu et al. (2024)^54^ studied the application of a Cu/FeOx composite catalyst for the dual purpose of upgrading heavy oil and promoting the low-temperature water–gas shift (LTWGS) reaction. Oxalic acid was utilized to generate the reactants necessary for the LTWGS reaction through its decomposition at 200 °C during the heavy crude oil upgrading process. Within the Cu/FeO_x_ composite, Cu⁰ catalyzed the LTWGS reaction, whereas α-Fe_2_O_3_ acted as the catalyst for heavy crude oil cracking. The in-situ H_2_ produced from the LTWGS reaction facilitated the catalytic reduction of heavy crude oil viscosity by preventing the recombination of radicals.

Previous reviews and studies have demonstrated that metal-based nanoparticles, including single-metal and multi-metal combinations, significantly enhance the upgrading of heavy crude oil. These catalysts exhibit high efficacy in reducing the viscosity of heavy oil due to their multifunctional properties, which include nanoscale size, high catalytic activity, large specific surface area, and efficient thermal conductivity. However, challenges persist in their preparation and application. These nanoparticles are prone to agglomeration and oxidation during synthesis, and their surfaces are difficult to modify. Moreover, they tend to aggregate and agglomerate during use, leading to inadequate dispersion and reduced effectiveness^55,56^. To address these challenges, loading nanoparticles onto carriers represents a promising solution. With their high specific surface area, thermal stability, and unique chemical properties, carriers help mitigate issues of aggregation and agglomeration. This approach not only improves the stability and dispersion of the metal nanoparticles but also enhances their overall catalytic performance^57–59^. One effective carrier material is graphene oxide (GO), which offers several advantages, including a large specific surface area, high thermal conductivity, and good chemical stability. Its surface is functionalized with polar oxygen-containing groups, such as hydroxyl, epoxy, and carboxyl groups, which help prevent the aggregation and leaching of metal nanoparticles^60^. Additionally, graphene oxide improves the dispersion of metal nanoparticles in heavy oil, enhancing their interaction with heavy oil molecules and thereby significantly boosting the catalytic performance of the composite for more effective upgrading and viscosity reduction. Consequently, GO-based metal nanocatalysts achieve a balance of high surface area and catalytic activity (from the homogeneous aspect), with high stability and effective dispersion (from the heterogeneous aspect)^61^.

In addition to enhancing catalytic performance, the magnetic properties of the metal nanocatalysts facilitate their separation and collection with minimal operational complexity, offering significant advantages for recovery and reuse. Magnetic separation is particularly well-suited for this application due to its simplicity, high efficiency, and rapid operation. Following catalytic reactions within the reservoir, the magnetic nanocatalysts can be efficiently recovered by applying an external magnetic field^51,62,63^. Surface equipment for this process includes a magnetic separator, which utilizes high-gradient magnetic coils or a magnetic drum to attract and capture the magnetic catalysts from the oil mixture. The recovered catalysts are then collected in a series of collection tanks or hoppers. Following separation, the catalysts are mixed with a carrier fluid and reintroduced into the reservoir via an injection pump and specialized injection wells^64^. This comprehensive approach not only simplifies the catalyst recovery process but also enhances the recyclability and reuse of the catalysts, thereby improving both economic and environmental sustainability. By implementing this technique, the integrity of the reservoir and the quality of the produced oil are preserved, while effectively managing catalyst resources.

In this study, the viability of in-situ upgrading of heavy crude oil using steam injection was evaluated through the application of two novel catalysts. The catalysts investigated were magnetic copper-substituted zinc ferrite (ZCFO) and a graphene oxide-based nanocomposite (GO-ZCFO) at different weight ratios (30:70, 50:50, and 70:30). The performance of these catalysts was experimentally assessed using a high-pressure/high-temperature reactor under controlled conditions, with a reaction temperature set at 320 °C and a reaction time of 6 h. A comprehensive analysis of the physical and chemical properties of the heavy oil before and after the upgrading process was conducted to determine the degree and extent of oil upgrading. This analysis included measurements of sulfur content, SARA fractions, viscosity, API gravity, and gas chromatography (GC) for saturated hydrocarbons and evolved gases.

## Materials and experimental setup

### Heavy oil

The heavy crude oil utilized in this investigation was sourced from an oil field located in the Western Desert of Egypt. Table 1 presents the chemical and physical characteristics of this original heavy crude oil.

**Table 1:** Chemical and physical properties of original heavy crude oil.

Properties, unit	Values
Density @ 15.56 °C, (g/ml)	0.9703
API Gravity @ 15.56 °C, (°)	14.24
Viscosity @ 40 °C, (mPa^.^s)	1160.81
Sulfur Content, (wt.%)	5.117
SARA Fractions, (wt.%)	
Saturates	26.98
Aromatics	39.40
Resins	17.77
Asphaltene	15.85

### Catalyst preparation

#### Preparation of graphene oxide (GO)

**Fig. 1:**
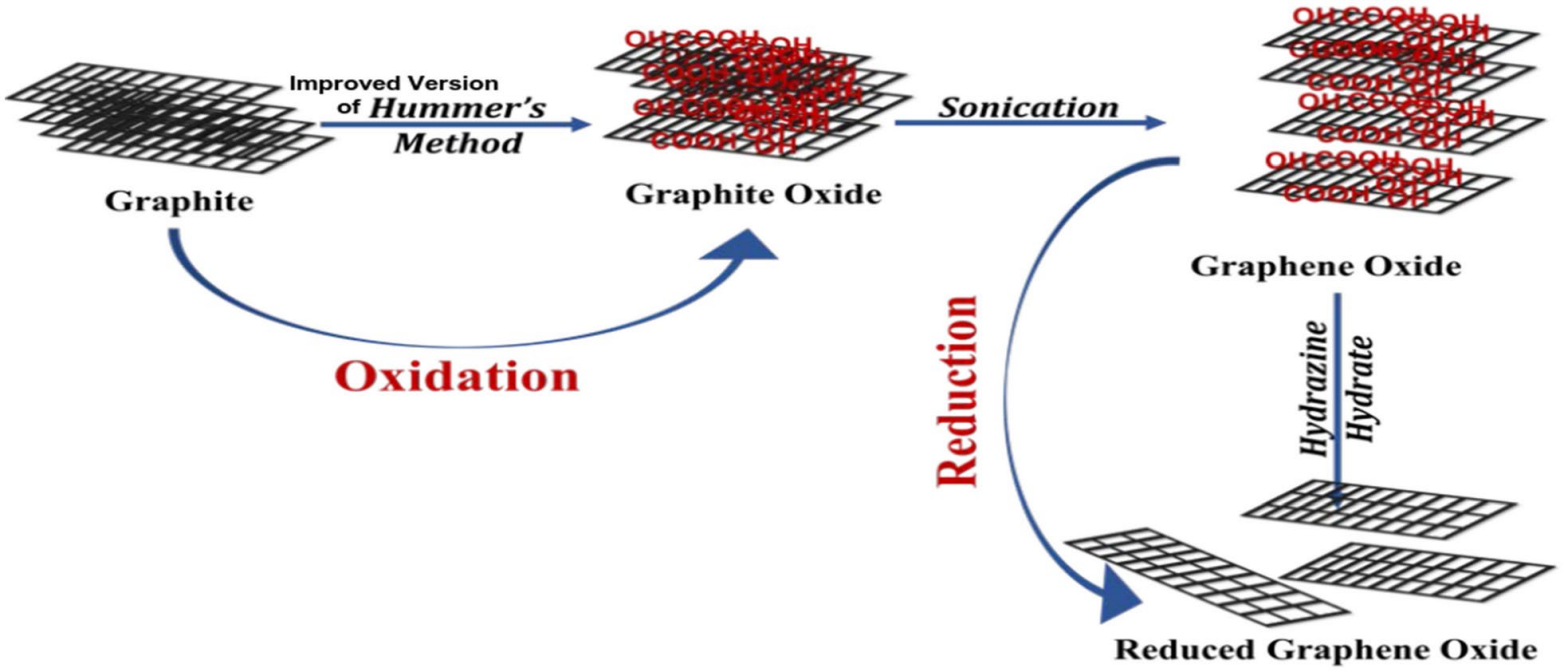
Schematic representation of the synthesis of reduced graphene oxide from graphite.


The material, graphene oxide (GO), was prepared from natural graphite flakes by using an improved version of Hummer’s method^65^ as shown in Fig. 1. This involved introducing a combination of concentrated H_2_SO_4_/H_3_PO_4_ (in a ratio of 9:1, totalling 360:40 ml) to a mixture of small flakes of graphite (3.0 g, equivalent to 1 weight) and KMnO_4_ (18.0 g, equivalent to 6 weights) resulting in a slight exothermic reaction within the temperature range of 35 to 40 °C. The mixture was then stirred for a duration of 12 h at a temperature of 50 °C, subsequently cooled to room temperature and poured into a combination of 400 ml of ice and 30% hydrogen peroxide H_2_O_2_ (3 ml). As a part of the preparation procedures, the mixture was subjected to sieving using a metal U.S. Standard testing sieve (W.S. Tyler, 300 μm) and filtration using polyester fiber (Carpenter Co.). The supernatant, resulting from centrifugation of the filtrate at 4000 rpm for 4 h, was decanted away. The residual solid substance was subsequently washed using water (200 ml), followed by HCl (200 ml, 30%), and ethanol (200 ml, 2X). For every washing cycle, the mixture was passed through the U.S. Standard testing sieve, followed by filtration using polyester fiber. The resulting filtrate underwent centrifugation at 4000 rpm for 4 h, and the supernatant was then carefully decanted. Following the prolonged and repeated washing process, the residual substance was coagulated by using ether (200 ml). The resulting suspension was subjected to filtration through a PTFE membrane, and the solid collected on the filter was dried under vacuum at room temperature overnight, resulting in the production of graphene oxide (GO). The chemical reaction can be represented as follows:

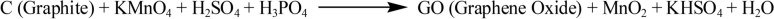


#### Preparation of copper zinc ferrite nano-catalyst (ZCFO)

Using a sol–gel combustion method, a magnetic copper substituted zinc ferrite nano-catalyst (ZCFO) was synthesized. The synthesis process was adapted from previous studies^66,67^. To synthesize the catalyst, a mixture of 2.97 g Zn(NO_3_)_2_**∙** 6H_2_O, 0.965 g Cu(NO_3_)_2_**∙** 3H_2_O, and 4.85 g Fe(NO_3_)_3_**∙** 9H_2_O was prepared in deionized water with a Cu/(Cu + Fe) ratio of 0.2. Following this, 6.30 g of citric acid was added to the mixture, and then the pH was adjusted to 5.4 using ammonium hydroxide. The resulting solution was agitated for a period of 2 h at a temperature of 60 ℃ and then heated to 90 ℃ for the purpose of evaporating the water content. The dried mixture was subjected to calcination at a temperature of 400 °C for a period of 2 h. This would cause the citric acid to decompose. Upon cooling to room temperature, the product was washed with 0.1 M of H_2_SO_4_, deionized water, and ethanol in alternating cycles. Lastly, it was dried in an oven at a temperature of 80 ℃ overnight. The overall process involves the following key steps and reactions:


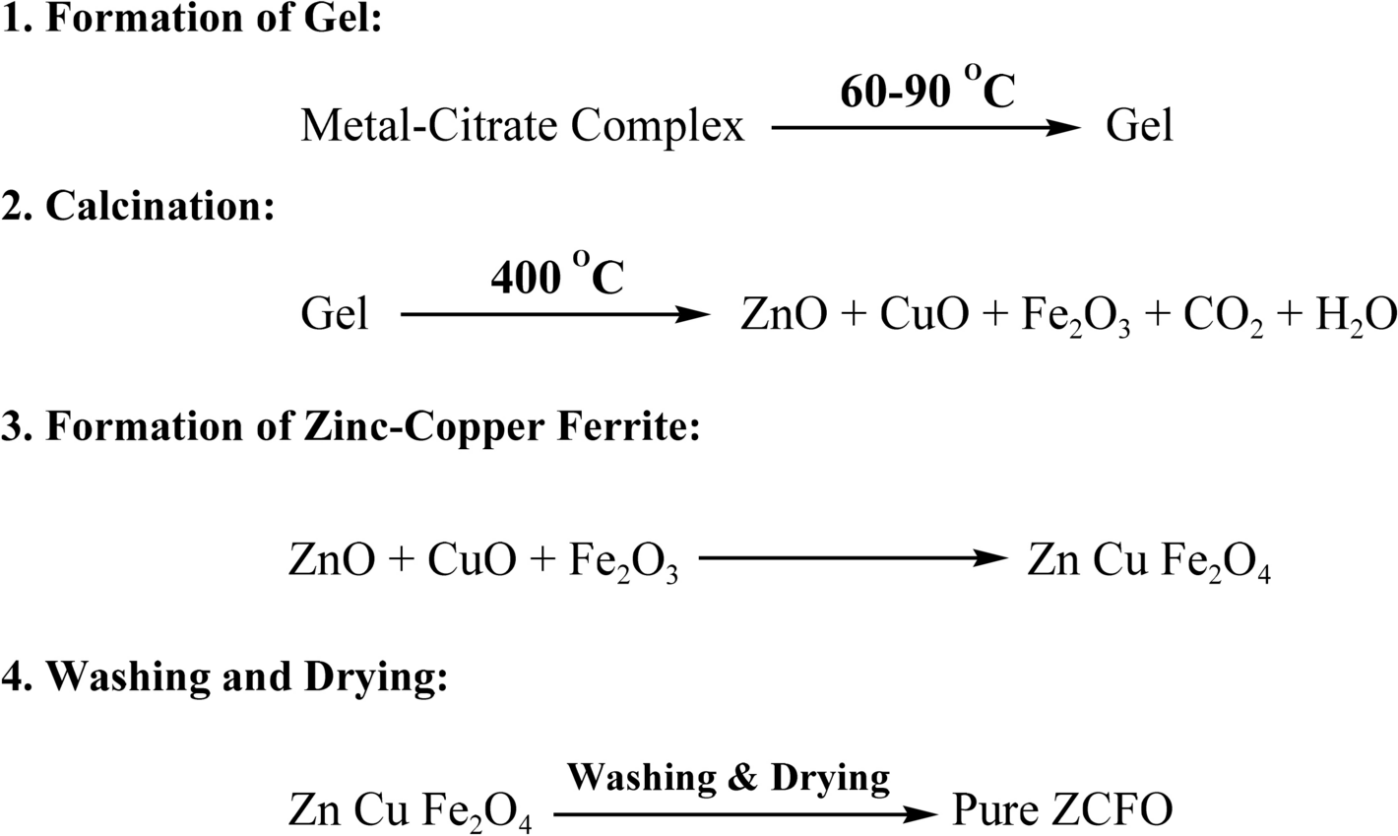


#### Preparation of nanocomposite (GO-ZCFO)

To synthesize a 50:50 weight ratio of graphene oxide-copper zinc ferrite nanocomposite (GO-ZCFO), 2.5 g of graphene oxide (GO) was dispersed in 100 ml of ethanol, followed by the addition of 2.5 g of copper zinc ferrite (ZCFO). The mixture was then stirred with sonication for an hour to ensure proper mixing. Subsequently, the resulting homogeneous solution was dried overnight at 80 °C to obtain the final powder. The preparation process can be summarized as follows:


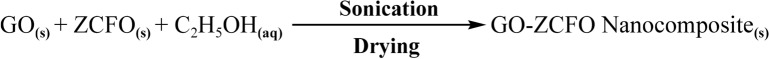


To investigate the potential of the GO-ZCFO nanocomposite in the in-situ upgrading of heavy crude oil, the weight ratio of GO to ZCFO was systematically varied to produce nanocomposite materials with weight ratios of 70:30 and 30:70.

### Catalyst characterization

#### X-ray diffraction analysis (XRD)

In this study, the crystallographic properties of the catalysts were investigated by using a powder X-ray diffractometer (Panalytical Empyrean 3 instrument from Malvern, Netherlands). The XRD measurements were conducted using a continuous scan approach, covering a 2θ angle ranging from 5.0100° to 90.00°, with a step size of 0.020° per 2θ and a measurement time of 0.50 s per step. Copper (Cu) was employed as the anode material for generating X-rays, resulting in the emission of K-Alpha1, K-Alpha2, and K-Beta wavelengths at 1.54060 Å, 1.54443 Å, and 1.39225 Å, respectively. The K-Alpha2 to K-Alpha1 wavelength ratio was 0.50000. To ensure accuracy and reliability, the XRD analysis was performed at a controlled temperature of 25 °C, and spinning was incorporated during measurements. The valuable insights obtained from this XRD analysis significantly contributed to a comprehensive understanding of the crystal structure and composition of the developed catalysts.

#### Fourier-transform infrared spectroscopy (FTIR)

Fourier Transform Infrared Spectroscopy (FTIR) served as an analytical technique to investigate the chemical composition and identify functional groups present in the catalysts. The instrument used for this analysis was the Vertex 70 RAM II, renowned for its high-performance capabilities. The mode of measurement adopted was ATR-FTIR (Attenuated Total Reflection—Fourier Transform Infrared), a non-destructive method that allows for direct analysis of solid and liquid samples without the need for extensive sample preparation. The FTIR measurements covered a broad spectral range from 4000 to 400 cm^-1^, allowing for the detection of various chemical functional groups in the samples. Additionally, the instrument’s spectral resolution of 4 cm^−1^ ensured precise and accurate spectral data, facilitating a comprehensive analysis of the chemical composition and molecular structure of the developed catalysts. The combination of the Vertex 70 RAM II model and ATR-FTIR proved highly effective in characterizing the samples, providing valuable insights for this study.

#### Transmission electron microscopy (TEM)

Transmission Electron Microscopy (TEM) was employed to investigate the internal structure and morphology of the catalysts used in the study. The analysis was performed using the Talos F200i (Thermo Fisher Scientific), a field emission (scanning) transmission electron microscope operating within a voltage range of 20–200 kV, renowned for its advanced high-resolution imaging capabilities. TEM allows for the observation of structural details at the atomic level, providing critical insights into the size, shape, and arrangement of catalytic particles.

### Heavy oil upgrading process

In our investigation, a high-pressure/high-temperature reactor was utilized for the in-situ upgrading of heavy crude oil through both catalytic and non-catalytic aquathermolysis. The reactor was equipped with specifications specifically tailored to meet the objectives of the study.

#### Characteristics of the experimental reactor

**Fig. 2:**
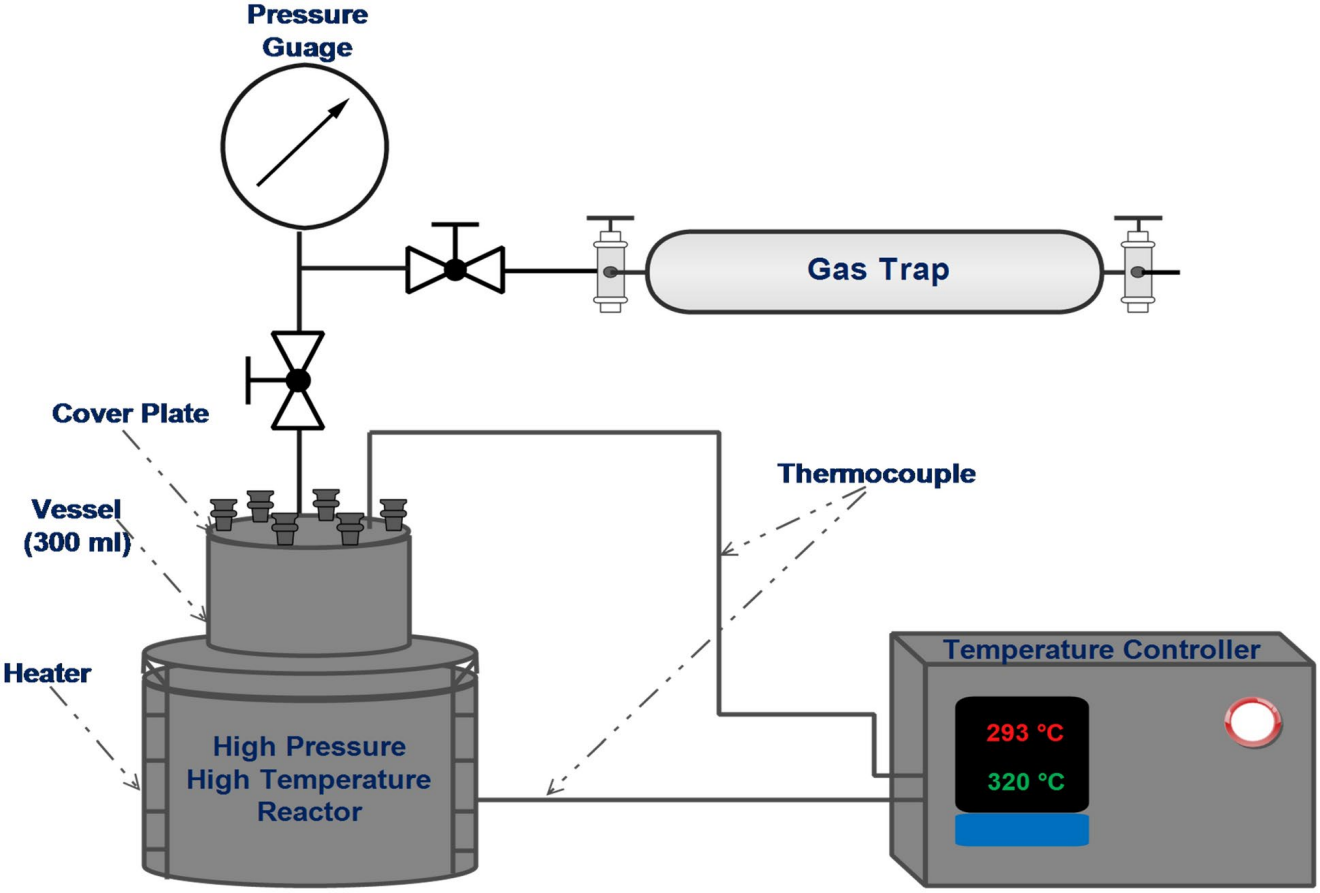
Schematic diagram of the high-pressure/high-temperature reactor for catalytic and non-catalytic upgrading processes.

The reactor, with a 300 ml volume, effectively accommodates the reaction mixture and catalyst. It operates within a temperature range of up to 400 °C and pressures of up to 250 bar, enabling the simulation of real operating conditions and optimization of the upgrading process. Additionally, the reactor is connected to a gas trap system, which plays a crucial role in capturing and collecting the evolved gases resulting from the upgrading process for subsequent compositional analysis. The combination of these specifications provides a robust and versatile system for conducting the in-situ upgrading study effectively. Figure 2 illustrates the schematic diagram of the reactor.

#### Experimental procedure

In the non-catalytic aquathermolysis experiment, a precise mixture of 70 g of heavy oil and 30 g of distilled water was carefully loaded into the reactor. For the catalytic aquathermolysis experiments, the reactor was loaded with oil, distilled water, and catalyst in a mass ratio of 70:30:0.15 g, respectively. In both experiments, the reactor was heated at a rate of 10 °C/min until reaching a temperature of 320 °C, which was then maintained for approximately 6 h. The selection of 320 °C as the reaction temperature is based on previous studies. According to Hyne (1986)^68^, significant thermal cracking occurs only at temperatures exceeding 300 °C, necessitating higher temperatures for efficient cracking. Additionally, Shakirullah et al. (2010)^69^ identified 320 °C as the optimal temperature for hydrodesulfurization, where the desulfurization yield reaches its maximum. Therefore, this temperature was chosen to maximize both thermal cracking and hydrodesulfurization efficiency during the aquathermolysis process. During the aquathermolysis process, gases were generated within the reactor, leading to a notable increase in the final pressure, which ranged from approximately 86 to 112 bar. This pressure elevation can be attributed to the accumulation of the produced gases. At the end of the heating period, the reactor was gradually cooled to room temperature, and the evolved gases were captured and collected using a gas trap system. The experimental conditions for both catalytic and non-catalytic aquathermolysis processes are summarized in Table 2.

**Table 2:** Experimental conditions for catalytic and non-catalytic aquathermolysis processes.

Exp No		Oil weight	Water weight	Catalyst weight	Type of catalyst	Reaction temperature	Final pressure	Reaction time
		(g)	(g)	(g)		(°C)	(bar)	(hr)
Non-catalytic aquathermolysis process	1	70	30	_	_	320	86	6
Catalytic aquathermolysis process	2	70	30	0.15	ZCFO	320	97	6
	3	70	30	0.15	GO- ZCFO (30:70)	320	111	6
	4	70	30	0.15	GO- ZCFO (50:50)	320	112	6
	5	70	30	0.15	GO- ZCFO (70:30)	320	109	6

### Analysis of heavy crude oil before and after the upgrading process

**Fig. 3:**
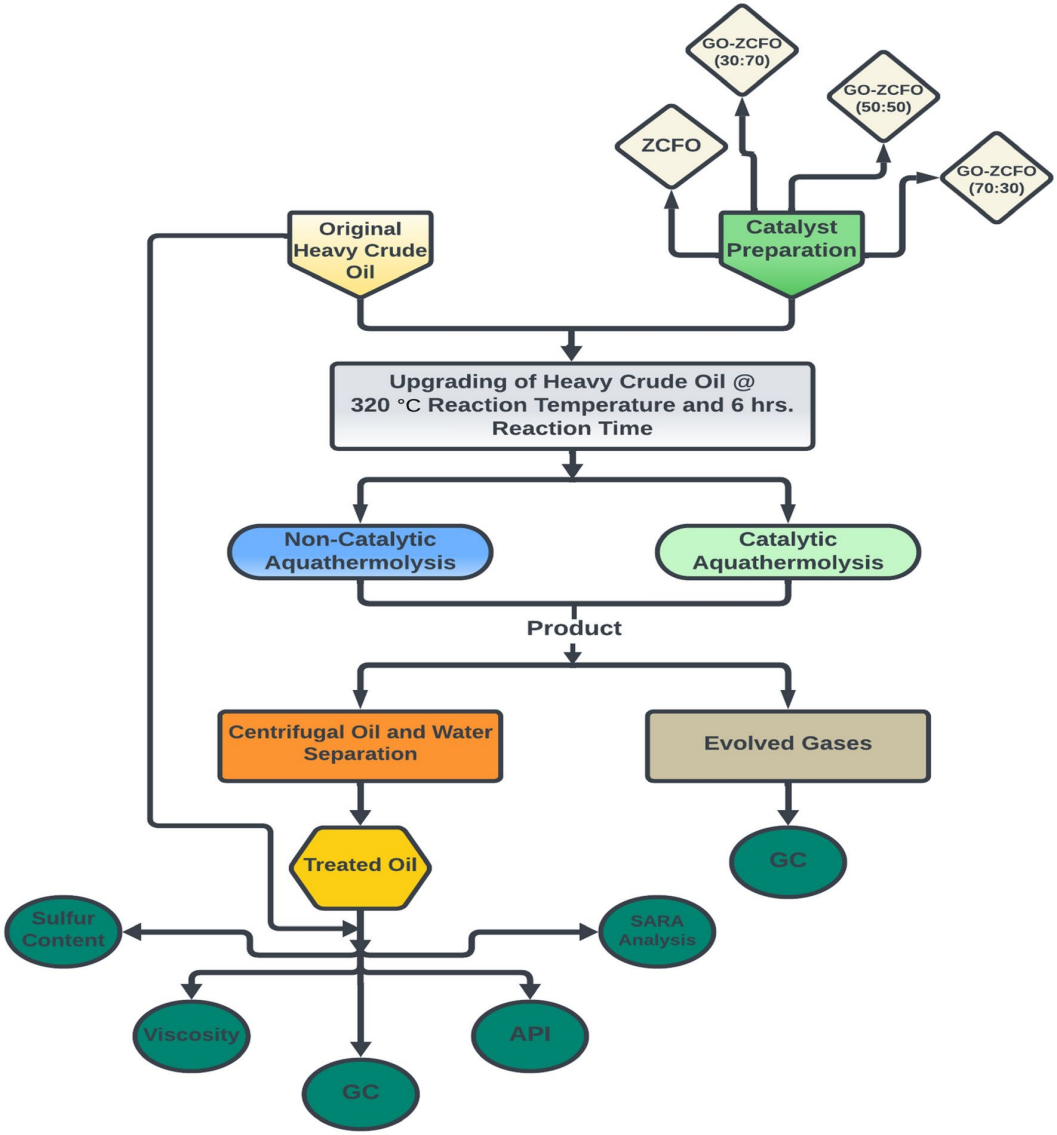
Schematic diagram of the catalytic and non-catalytic upgrading processes, along with the techniques for analyzing the resulting products.

In the pursuit of a comprehensive understanding of heavy crude oil properties and their changes in both physical and chemical characteristics before and after upgrading processes, a series of analytical techniques were employed. As depicted in Fig. 3, a schematic diagram illustrates both catalytic and non-catalytic upgrading processes, along with the techniques used to analyze the resulting products.

#### API gravity analysis

API gravity was measured at a temperature of 15.56 °C, utilizing the Anton Paar SVM 3001, a highly regarded instrument renowned for its accuracy and precision. This measurement was carried out in strict conformity with the ASTM D-4052 methodology, ensuring accuracy and reliability in the obtained results.

#### Viscosity analysis

Heavy oil viscosity was measured at a precisely controlled temperature of 40 °C, using the Anton Paar SVM 3001 Stabinger Viscometer. This measurement adhered to the ASTM D-445 standard, underscoring the credibility and consistency of the obtained results.

#### Sulfur content analysis

Sulfur content in heavy crude oil was analyzed following ASTM D-4294 guidelines using a Spectroscan-S analyzer equipped with X-ray fluorescence (XRF) technology. This instrument provided rapid and highly accurate measurements of sulfur content.

#### SARA analysis

The determination of asphaltene content in heavy crude oil was carried out following the IP 143/90 method outlined in the IP Standards for Analysis and Testing of Petroleum and Related Products from 1993^70^. The procedure involved several steps: First, a mixture of heavy crude oil and a paraffinic solvent, n-heptane (at a ratio of 30 ml per 1 g of crude oil), was prepared. This mixture was then subjected to heat under reflux for a duration of 2 h. After refluxing, the flask was allowed to cool in a dark place. The resulting residue was transferred as completely as possible to a filter paper, which was subsequently placed into a Soxhlet extractor. Refluxing with n-heptane continued until the condensed n-heptane was colorless, indicating the complete extraction of soluble components. Excess solvent was removed through distillation, and the resulting maltenes were dried to a constant weight. Following this, the asphaltene retained on the filter paper was subjected to reflux with benzene until all the asphaltene was completely dissolved. The dissolved asphaltene was then dried until a constant weight was achieved. After determining the asphaltene content, the maltenes were fractionated into saturated hydrocarbons, aromatic compounds, and resins using a chromatographic column packed with an adsorbent, as per the ASTM D4124 method^71^.

#### GC for saturated hydrocarbons

The investigation involved the analysis of n-alkane disruption in the saturated hydrocarbons of heavy crude oil, both before and after upgrading, utilizing an Agilent 7890A gas chromatograph. This gas chromatograph, equipped with a Flame Ionization Detector (FID), featured a capillary column measuring 30 inches in length and 0.32 mm in diameter. Nitrogen served as the carrier gas, flowing at a rate of 1.5 ml/min. The injector and detector temperatures were set at 300 °C and 320 °C, respectively. The column temperature underwent a gradual increase from 60 °C to 300 °C at a rate of 5 °C per minute, followed by an isothermal phase at 320 °C until the completion of the analysis. Compound identification was achieved by integrating peak areas using the Agilent ChemStation software.

#### GC for evolved gases

The analysis of gas samples for detecting hydrocarbons from C_1_ to C_9_, along with nitrogen, hydrogen, hydrogen sulfide, and carbon dioxide was conducted using a SCION 456-GC Natural gas analyzer. This analyzer is equipped with a set of columns and incorporates two detectors, namely the Flame Ionization Detector (FID) and Thermal Conductivity Detector (TCD). The analysis of light hydrocarbons (C_1_–C_9_) was performed using a capillary column, SCION-1, with a length of 60 m, an internal diameter of 0.25 mm, and a film thickness of 1.0 µm, connected to the FID. The analysis of nitrogen, hydrogen, hydrogen sulfide, and carbon dioxide was conducted using packed columns: Molsieve 13X (80/100 mesh size, 1.5 m in length, and 1/8-inch internal diameter) and Hayesep Q (80/100 mesh size, 0.5 m in length, and 1/8-inch internal diameter). The TCD was connected to both packed columns, with helium used as the carrier gas. The elution of the examined gas mixture was accomplished through temperature programming, progressing from 50 to 160 °C at a rate of 6 °C/min. Quantitative analysis was performed by utilizing a standard natural gas sample with a known composition, adhering to the [ASTM: D 1945–03] standard. Injector and detector temperatures were set at 200 °C and 250 °C, respectively. Data analysis involved the estimation of values through the integration of the area under the resolved chromatographic profiles, utilizing the Compass CDS software on the HP computer.

## Results and discussion

### Catalysts’ characterization through XRD analysis

**Fig. 4:**
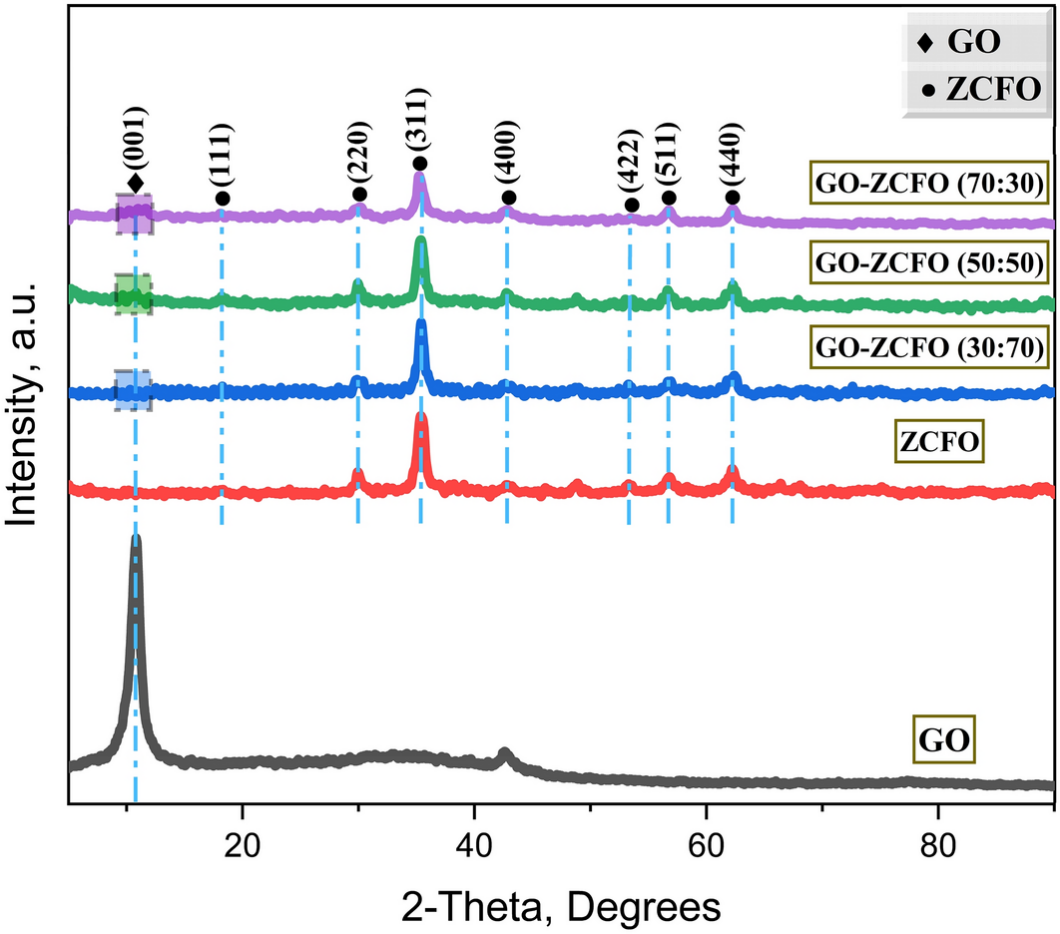
XRD powder patterns of graphene oxide, magnetic copper substituted zinc ferrite (ZCFO), and graphene oxide-based nanocomposite (GO-ZCFO) with different weight ratios (30:70), (50:50), and (70:30).

The X-ray diffractometer was employed to examine the catalysts’ phase purity and crystal structure. Figure 4displays the XRD spectrum of GO, ZCFO, and GO-ZCFO nanocomposites with different ratios. In the XRD spectrum of GO, the characteristic peak of the GO sheet attributed to (001) appeared at 2-Theta = 10.75°, which corresponds to a definite d-spacing of 0.82 nm^65^. In the XRD spectrum of ZCFO, a set of distinctive diffraction peaks were observed at 18.19°, 30°, 35.40°, 42.86°, 53.34°, 56.74°, and 62.17°, corresponding to the (111), (220), (311), (400), (422), (511), and (440) crystal planes of cubic spinel zinc ferrite, as identified by the ICDD card with reference code (01–083-3927)^72^. It is noteworthy that the spectrum showed no detectable peaks corresponding to copper oxides, specifically Cu_2_O or CuO, demonstrating that Cu was effectively integrated into the ZnFe_2_O_4_ lattice without influencing its spinel structure. In the XRD spectrum of GO-ZCFO with different ratios, the GO-ZCFO nanocomposite exhibited all the peaks of both GO and ZCFO. However, as the loading of ZCFO onto the GO sheets increased, the intensity of the GO peak at 2-Theta = 10.75° gradually decreased and eventually disappeared in the XRD spectrum of GO-ZCFO with a weight ratio of (30:70), as shown in Fig. 4. This observation reveals the strong interaction of ZCFO with GO sheets. Moreover, the strong intensity of the diffraction peaks suggests that both pure ZCFO and GO-ZCFO are well crystallized.

### Catalysts’ characterization through FTIR analysis

**Fig. 5:**
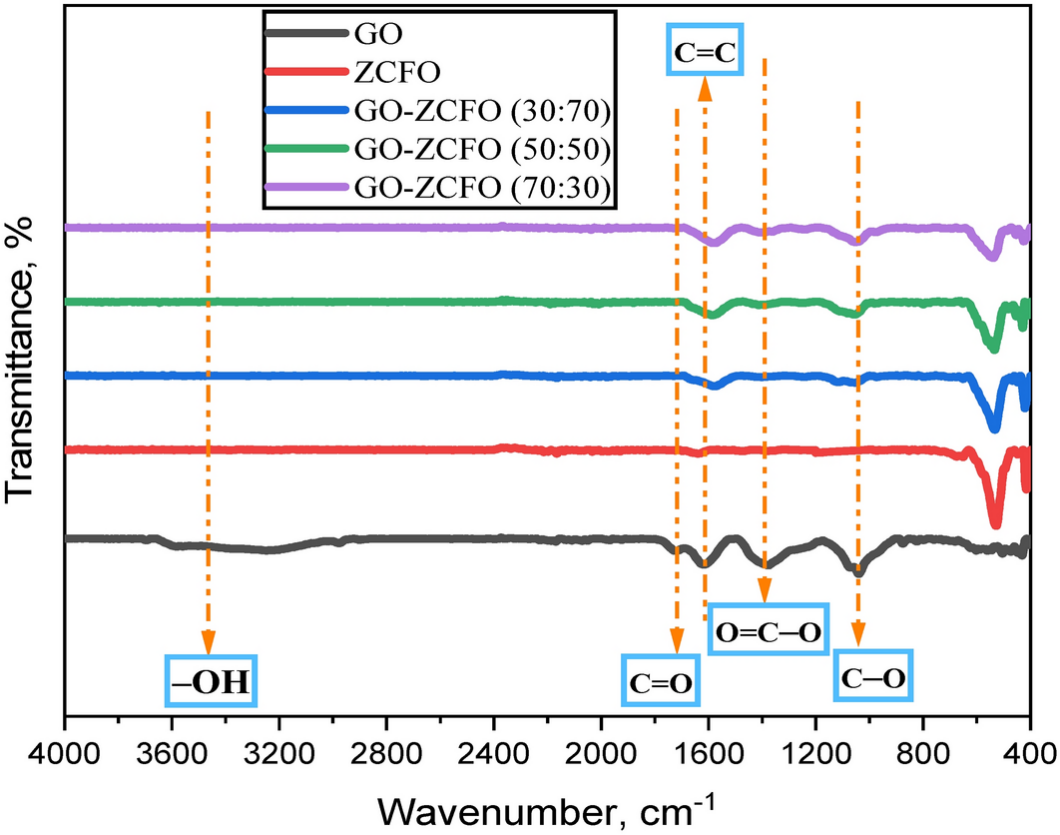
FTIR spectra of graphene oxide, magnetic copper substituted zinc ferrite (ZCFO), and graphene oxide-based nanocomposite (GO-ZCFO) with different weight ratios (30:70), (50:50), and (70:30).

FTIR spectra were recorded in the wavenumber region of (4000–400) cm^-1^ to investigate the efficient incorporation of ZCFO nanoparticles into the GO matrix and to identify various functional groups in the nanocomposite catalyst. The FTIR spectra of GO, ZCFO, and GO-ZCFO with different weight ratios are depicted in Fig. 5. In the spectrum of GO, the broad absorption peak (band) between 3200 and 3500 cm^−1^ is associated with the stretching vibration of OH groups, revealing the presence of hydroxyl groups in the graphene oxide sheet. The peak (band) observed at 1725 cm^−1^ is attributed to the C = O stretch of the carboxylic groups, while the sharp peak found at 1614 cm^−1^can be assigned to aromatic (C = C) bonds of un-oxidized graphitic domains^73^. The peak located at 1402 cm^−1^ corresponds to the O = C − O stretch of carboxylic groups attached to the graphene-conjugated system. Whereas the peak at 1038 cm^−1^arises from the vibrational mode of the C − O stretch. These observations align well with what has been reported in previous literature^65,74,75^. In the spectrum of ZCFO, vibrations of ions within the crystalline lattice are typically detected over the 400–1000 cm^-1^ range. In general, spinel ferrites exhibit two absorption bands associated with metal–oxygen (MO) interactions, which occur at both tetrahedral and octahedral sites. The metal–oxygen (MO) band is characterized by a higher frequency at the tetrahedral site, while the band at the octahedral site has a lower frequency. As depicted in Fig. 5, two distinct absorption peaks were detected at approximately ∼551 and ∼427 cm^−1^, which could be ascribed to the intrinsic stretching vibrations of the Zn–O at the tetrahedral site and Fe–O at the octahedral site, respectively^76,77^. In the spectrum of the GO-ZCFO nanocomposite, the peaks corresponding to the distinct functional groups of ZCFO and GO were slightly shifted to lower wavenumbers with noticeable weakening. This alteration is attributed to the robust interaction between these groups, indicating the effective binding of ZCFO nanoparticles on the active surfaces of GO sheets^78^. Besides, the distinctive broad peak within the 3200–3500 cm^-1^ range, representing the OH-stretching vibrations in GO, has nearly disappeared from the spectrum of GO-ZCFO nanocomposite with different weight ratios.

### Catalysts’ characterization through TEM analysis

**Fig. 6:**
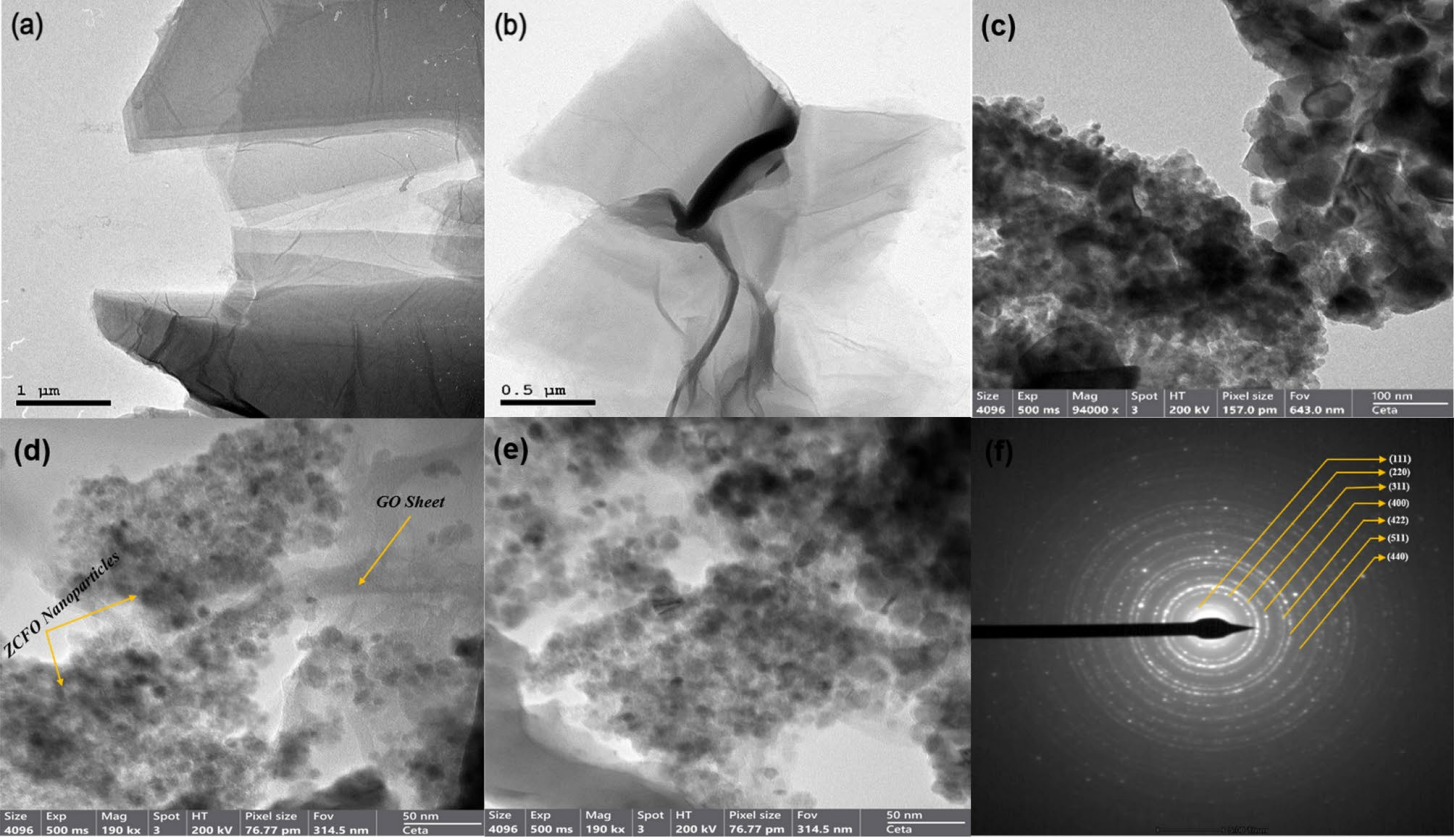
(a-b) TEM images of GO, (c-e) HR-TEM images of GO-ZCFO nanocomposite, and (f) SAED image of GO-ZCFO nanocomposite.

The TEM micrograph reveals that graphene oxide (GO) exhibits sheet-like structures with a deep brown color, which is characteristic of this material, as shown in Fig. 6(a-b). The sheets have significant lengths ranging from 1 to 3 µm, and their thickness varies between 1 and 5 nm, confirming the two-dimensional morphology and layered structure typical of graphene oxide. The high-resolution TEM (HR-TEM) images, as shown in Fig. 6(c-e), confirm the incorporation of ZCFO nanoparticles within the GO matrix. The crystalline nature of the ZCFO nanoparticles was initially verified by XRD analysis, as depicted in Fig. 4, and further supported by Selected Area Electron Diffraction (SAED) analysis, which reveals a ring-like pattern, as shown in Fig. 6(f). The observed rings correspond to the (111), (220), (311), (400), (422), (511), and (440) crystal planes of cubic spinel zinc ferrite, indicating that the GO-ZCFO nanocomposite maintains both the structural integrity of graphene oxide and the crystalline nature of the copper zinc ferrite phase. This unique combination of properties is expected to contribute to the enhanced functional characteristics of the nanocomposite.

### Catalyst effect on sulfur content of heavy oil

**Fig. 7:**
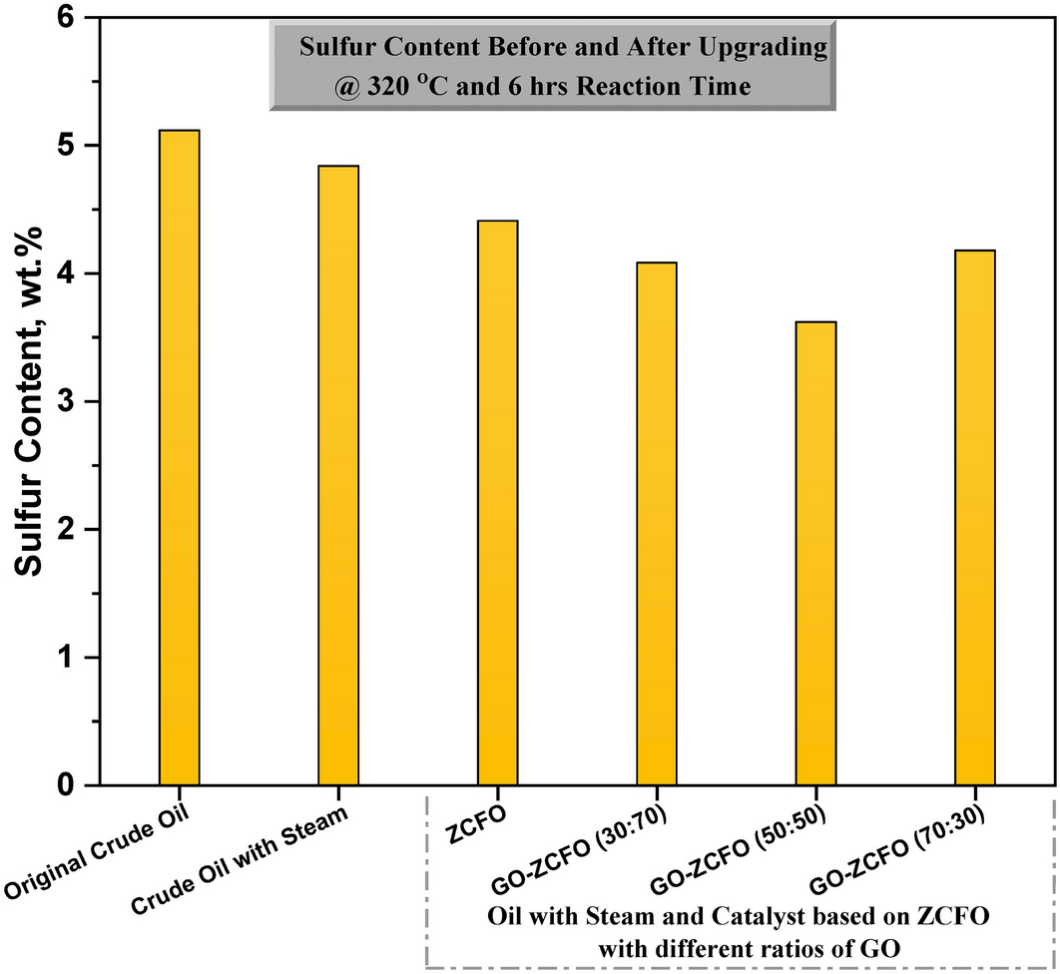
Sulfur content analysis in percentage before and after upgrading of heavy oil at 320 °C and 6 h reaction time.

Table 3demonstrates the effect of both catalytic and non-catalytic aquathermolysis processes on the sulfur content of heavy crude oil. A key characteristic of heavy crude oil is its high content of resins and asphaltenes, both of which consist of a complex mixture of high-molecular-weight components. The high viscosity of heavy crude oil can be attributed to the presence of resins and asphaltenes^79^. Asphaltenes have a molecular structure consisting of polyaromatic rings with aliphatic carbon chains, while resins are comprised mainly of fused aromatic rings with branched paraffin and polar compounds. Both resins and asphaltenes contain heteroatoms such as sulfur, nitrogen, and oxygen into their respective composition^80,81^. In heavy crude oil, the C–S bond, with an energy of 305 kJ/mol, is comparatively weaker than the C–C bond, which has an energy of 346 kJ/mol, facilitating the easier cleavage of C–S bonds during aquathermolysis reactions. Consequently, this process breaks down the high-molecular-weight components of resin and asphaltene into lighter constituents, leading to a reduction in viscosity^48,82,83^. As shown in Table 3, the original crude oil serves as a reference point with a sulfur content of 5.117 wt.%. When heavy crude oil underwent non-catalytic aquathermolysis, there was a slight reduction in sulfur content to 4.84 wt.%. However, with the introduction of catalysts, a significant decrease in sulfur content was observed due to the hydrodesulfurization of organo-sulfur compounds within resin and asphaltene molecules. Results indicated that employing ZCFO and GO-ZCFO catalysts with different weight ratios (30:70, 50:50, and 70:30) resulted in sulfur content reductions to 4.41 wt.%, 4.083 wt.%, 3.62 wt.%, and 4.18 wt.%, respectively. This notable reduction was facilitated by aquathermolysis catalysts based on Cu, Zn, Fe, and GO, which aid in lowering the energy barriers and catalyzing the cleavage of the C–S bond. As depicted in Fig. 7, the sulfur content analysis indicates that GO-ZCFO (50:50) exhibits the most effective catalytic performance in the hydrodesulfurization of high-molecular-weight components in heavy crude oil.

**Table 3:** Sulfur content analysis before and after upgrading of heavy oil at 320 °C and 6 h reaction time.

Sample		Sulfur content
		(wt.%)
Original crude oil		5.117
Crude oil with steam		4.84
Crude oil with steam and catalyst based on	ZCFO	4.41
	GO- ZCFO (30:70)	4.083
	GO- ZCFO (50:50)	3.62
	GO- ZCFO (70:30)	4.18

### Catalyst effect on SARA analysis of heavy oil

**Fig. 8:**
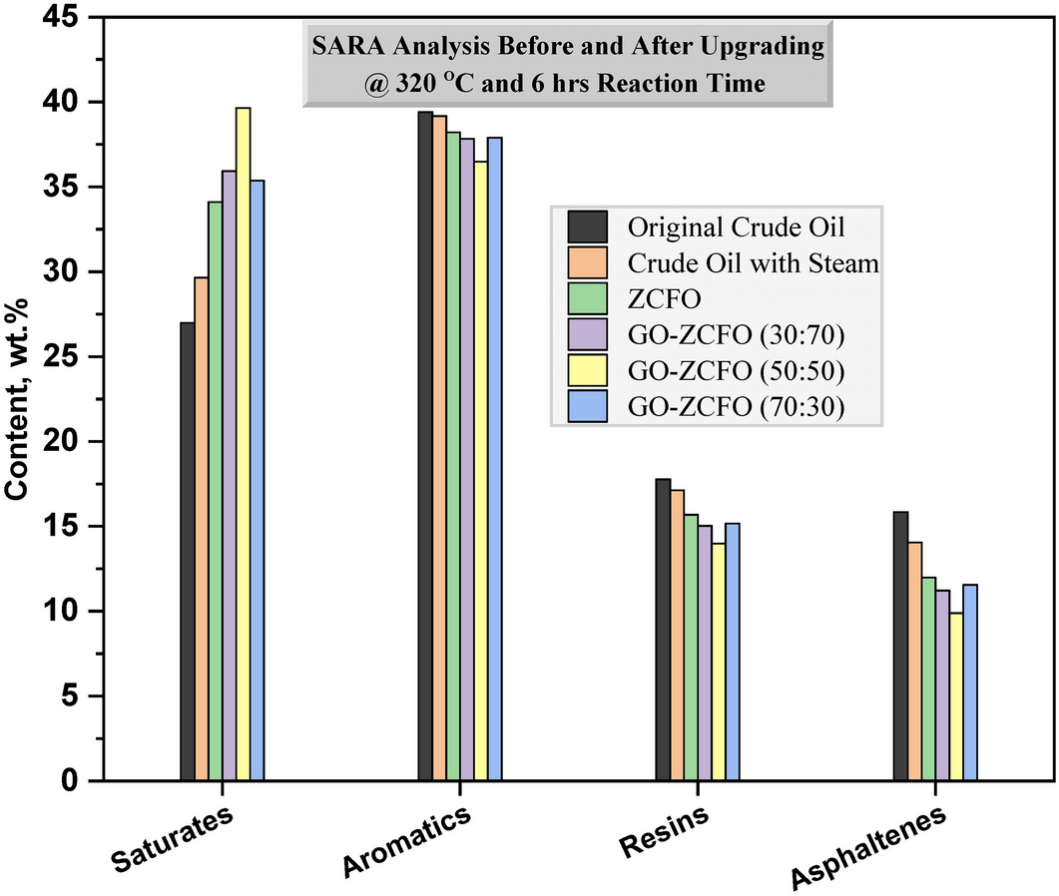
SARA analysis in percentage before and after upgrading process of heavy oil at 320 °C and 6 h reaction time.

The SARA analysis (Saturates, Aromatics, Resins, and Asphaltenes) for heavy crude oil is presented in Table 4, both before and after the upgrading process. The observed decrease in the sulfur content of the heavy crude oil signifies the cleavage of C–S bonds, which contributes to the reduction in resin and asphaltene content accompanied by an increase in saturated hydrocarbon content, as detailed in Tables 3 and 4. Initially, the original crude oil exhibits resin, asphaltene, and saturate contents of 17.77 wt.%, 15.85 wt.%, and 26.98 wt.%, respectively. When subjected to steam only at 320 °C, a reduction was observed, resulting in resin and asphaltene content of 17.13 wt.% and 14.05 wt.%, respectively, while the saturated hydrocarbon content increased to 29.65 wt.%. Significant changes, marked by notable decreases in resin and asphaltene content and increases in the saturated hydrocarbon content, were observed with the incorporation of catalysts into the aquathermolysis process. As illustrated in Fig. 8, the resin content in the upgraded oil samples, achieved through catalytic aquathermolysis, was reduced to 15.68 wt.%, 15.03 wt.%, 13.99 wt.%, and 15.17 wt.% for ZCFO, GO-ZCFO (30:70), (50:50), and (70:30), respectively. Similarly, the asphaltene content reduced to 11.99 wt.%, 11.21 wt.%, 9.89 wt.%, and 11.56 wt.% for ZCFO, GO-ZCFO nanocomposite with varying weight ratios (30:70), (50:50), and (70:30), respectively. Concurrently, the saturated hydrocarbon content increased to 34.11 wt.%, 35.92 wt.%, 39.64 wt.%, and 35.37 wt.% for ZCFO, GO-ZCFO (30:70), (50:50), and (70:30), respectively. These findings highlight the remarkable effectiveness of GO-ZCFO (50:50) in significantly reducing the resin and asphaltene content while increasing the saturated hydrocarbon content in heavy crude oil.

**Table 4:** SARA analysis before and after upgrading of heavy oil at 320 °C and 6 h reaction time.

Sample		SARA Analysis (wt.%)			
		Saturates	Aromatics	Resins	Asphaltenes
Original crude oil		26.98	39.40	17.77	15.85
Crude oil with steam		29.65	39.17	17.13	14.05
Crude oil with steam and catalyst based on	ZCFO	34.11	38.22	15.68	11.99
	GO- ZCFO (30:70)	35.92	37.84	15.03	11.21
	GO- ZCFO (50:50)	39.64	36.48	13.99	9.89
	GO- ZCFO (70:30)	35.37	37.90	15.17	11.56

### Catalyst effect on viscosity and API gravity of heavy oil

**Fig. 9:**
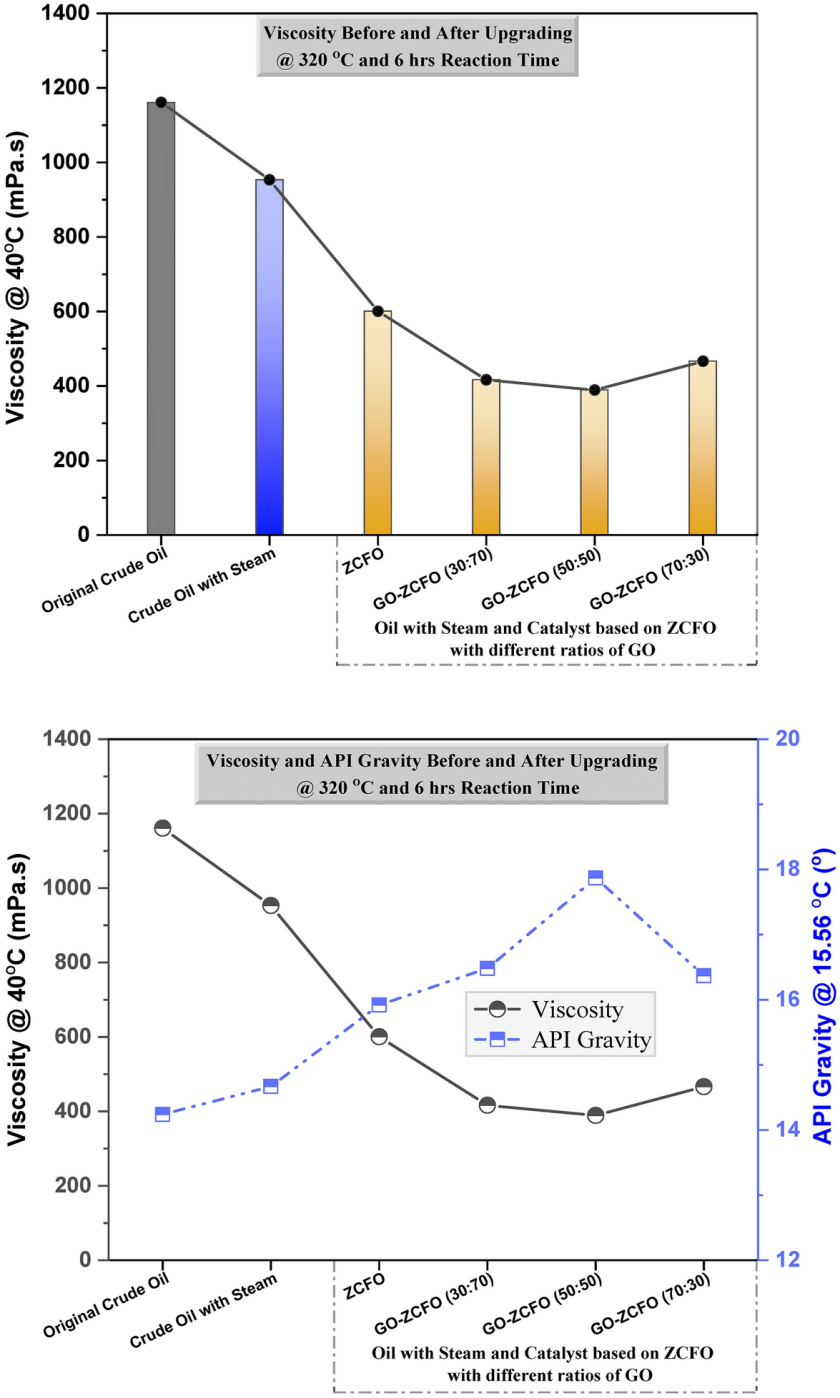
Viscosity and API gravity of the heavy oil before and after upgrading process at 320 °C and 6 h reaction time, measured at 40 °C and 15.56 °C, respectively.

As depicted in Table 5, the viscosity and API gravity of the heavy oil before and after the aquathermolysis process, with and without catalysts, were measured at temperatures of 40 °C and 15.56 °C, respectively. The observed reduction in sulfur content corresponds with a decrease in resin and asphaltene content, which subsequently leads to a reduction in the viscosity of heavy crude oil and an increase in its API gravity, as illustrated in Tables 3, 4, and 5. As shown in Fig. 9, non-catalytic aquathermolysis led to a slight decrease in the viscosity of heavy crude oil, dropping from 1160.81 to 953.16 mPa^.^s, accompanied by an increase in API gravity from 14.24° to 14.67°. The introduction of catalysts further enhanced these effects, reducing the viscosity of the upgraded oil by approximately threefold (from 1160.81 to less than 390 mPa^.^s, depending on the catalyst used) and increasing the API gravity from 14.24° to 17.87°. This indicates that these catalysts exhibit a notable catalytic effect in the aquathermolysis reaction. Among the catalysts tested, GO-ZCFO (50:50) displayed the best results in terms of sulfur content, saturated hydrocarbon content, resin and asphaltene content, viscosity, and API gravity.

**Table 5:** Viscosity and API gravity analysis before and after upgrading of heavy oil at 320 °C and 6 h reaction time.

Sample		Viscosity @ 40 °C	API gravity @ 15.56 °C
		(mPa.s)	(°)
Original crude oil		1160.81	14.24
Crude oil with steam		953.16	14.67
Crude oil with steam and catalyst based on	ZCFO	600.37	15.92
	GO-ZCFO (30:70)	416.22	16.48
	GO-ZCFO (50:50)	389.09	17.87
	GO-ZCFO (70:30)	466.41	16.37

### GC analysis of the saturated hydrocarbons before and after upgrading

**Fig. 10:**
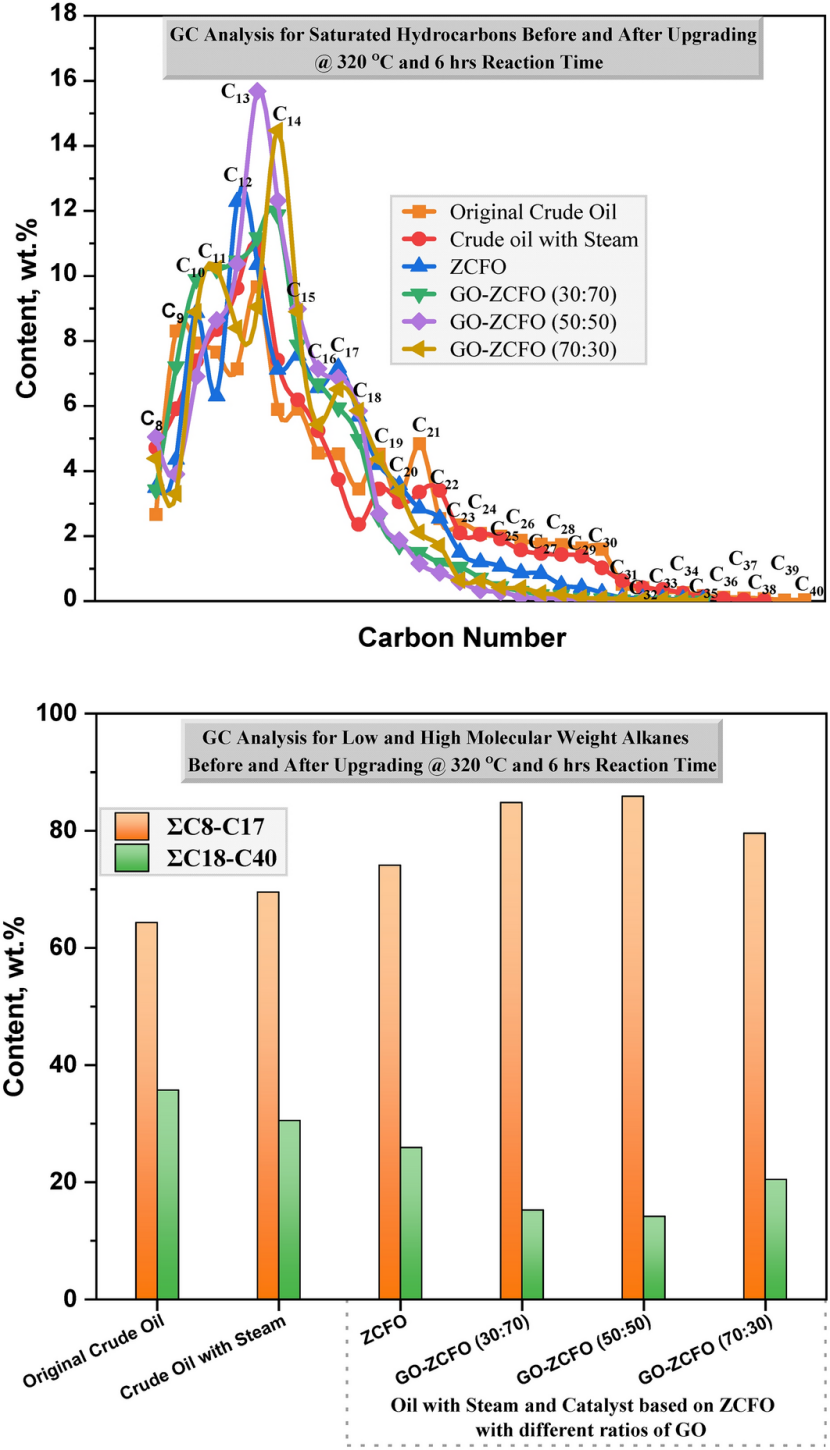
Composition of saturated hydrocarbons in the heavy oil before and after upgrading process at 320 °C and 6 h reaction time.

Figure 10 illustrates the gas chromatography (GC) results for the saturated hydrocarbons before and after the catalytic and non-catalytic aquathermolysis upgrading processes. The carbon number distribution and content in the saturated hydrocarbons, both before and after upgrading, are listed in **Table S-1** of the **Supplementary Material**. As shown in Fig. 10**,** the application of non-catalytic aquathermolysis for upgrading heavy crude oil resulted in minimal changes (slight increase) in the content of low-molecular-weight alkanes (C_8_–C_17_). In contrast, the introduction of catalysts into the aquathermolysis process led to a substantial modification (marked increase) in the content of low-molecular-weight hydrocarbons (C_8_–C_17_), accompanied by a decrease in the content of high-molecular-weight alkanes (C_18_–C_40_). These findings indicate that the presence of catalysts facilitates the breakdown of long-chain heavy hydrocarbon into lighter alkanes through the aquathermolysis reaction, which destructs the main structure of resin and asphaltene molecules. The formation of these lighter fractions contributes to reducing the viscosity of heavy crude oil. Based on the obtained results, GO-ZCFO (50:50) demonstrated the most effective catalytic performance, as evidenced by the significant increase in low-molecular-weight alkane components in the upgraded oil, as shown in Fig. 10.

### GC analysis of the evolved gases resulting from heavy oil upgrading

Table 6 presents the composition of the evolved gases resulting from the oil upgrading process, which includes both catalytic and non-catalytic aquathermolysis. During these reactions, the energy required for breaking the C–S bond in organo-sulfur compounds is minimal, leading to the generation of hydrogen sulfide (H_2_S), which serves as a reliable indicator of the degree or extent of aquathermolysis reactions. As depicted in Table 6, the chemical compounds (H_2_, CO_2_, H_2_S, and CH_4_) generated from aquathermolysis reactions confirm the cleavage of C–S bonds. The production of H_2_S during catalytic and non-catalytic aquathermolysis is predominantly attributed to the thermal and hydrolytic decomposition of organo-sulfur compounds within the resin and asphaltene molecules^84^. This implies that organo-sulfur compounds underwent a conversion process, resulting in the formation of either paraffinic or aromatic hydrocarbons, thus breaking down large heavy oil molecules into smaller ones^49^.

**Table 6:** Composition of evolved gases after upgrading heavy oil at 320 °C for 6 h.

Evolved Gases Composition, wt.%								
Samples		Gases			Saturated Hydrocarbons			Unsaturated Hydrocarbons
					Normal Alkane Gases		Iso Alkane Gases	Alkene
		H2	CO2	H2S	n(C1–C5)	C6+	i(C4–C5)	Olefins(C2–C5)
Original crude oil		_	_	_	_	_	_	_
Crude oil with steam		0.125	19.273	8.826	17.370	46.256	6.138	2.012
Crude oil with steam and catalyst based on	ZCFO	0.128	9.533	14.486	21.684	45.007	6.578	2.584
	GO-ZCFO (30:70)	ND	13.863	24.552	28.896	19.937	9.840	2.912
	GO-ZCFO (50:50)	0.283	22.989	34.256	37.308	0.344	4.241	0.579
	GO-ZCFO (70:30)	ND	18.136	20.875	26.596	21.885	10.110	2.398

**Fig. 11:**
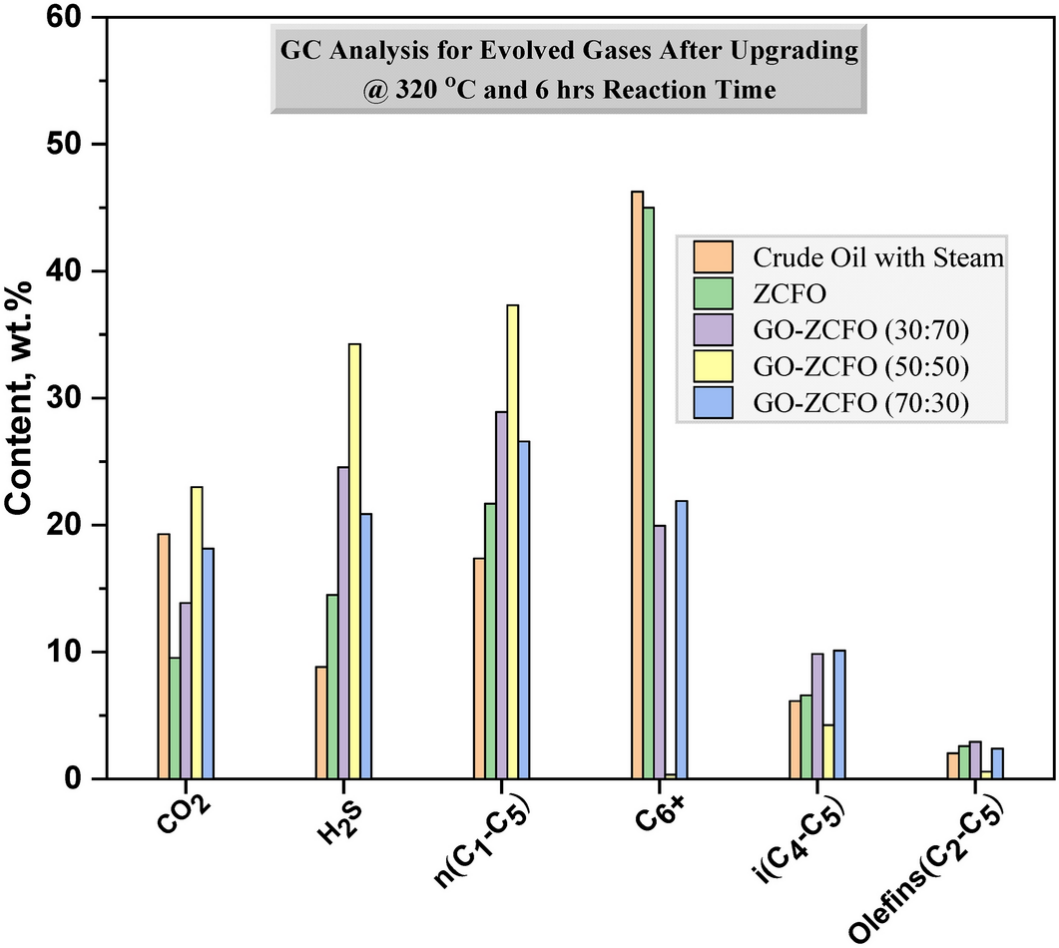
GC analysis of the evolved gases obtained after upgrading of heavy oil at 320 °C and 6 h reaction time.

As observed in Fig. 11, heavy crude oil subjected to steam treatment at 320 °C exhibits an H_2_S content of 8.826%, highlighting the influence of the non-catalytic aquathermolysis process on organo-sulfur compounds. The introduction of the ZCFO catalyst increases the H_2_S content to 14.486 wt.%, indicating the catalytic effect on hydrogen sulfide generation. Furthermore, a notable effect is observed with the incorporation of ZCFO nano-particles on the GO matrix in different ratios (30:70, 50:50, and 70:30). Specifically, the GO-ZCFO nanocomposite with weight ratio (50:50) demonstrates the highest H_2_S content, reaching 34.256 wt.%. The H_2_S generated during both catalytic and non-catalytic aquathermolysis aligns with the reduction observed in the sulfur content of the upgraded oil samples, as presented in Tables 3 and 6.

As outlined in Table 6, after both catalytic and non-catalytic aquathermolysis reactions, Gas Chromatography (GC) identifies another major gas, specifically carbon dioxide (CO_2_). The water–gas shift reaction (WGSR) is proposed as the main source of CO_2_generation^85^. As shown in Fig. 11, a discernible trend is observed wherein the presence of catalysts, particularly the GO-ZCFO (50:50) nanocomposite, leads to an increase in the total amount of light hydrocarbons n(C_1_–C_5_). This implies that the catalytic aquathermolysis process generates higher amounts of light hydrocarbon gases n(C_1_–C_5_) compared to the non-catalytic aquathermolysis process.

### Catalytic aquathermolysis: Reaction mechanisms and pathways

**Fig. 12:**
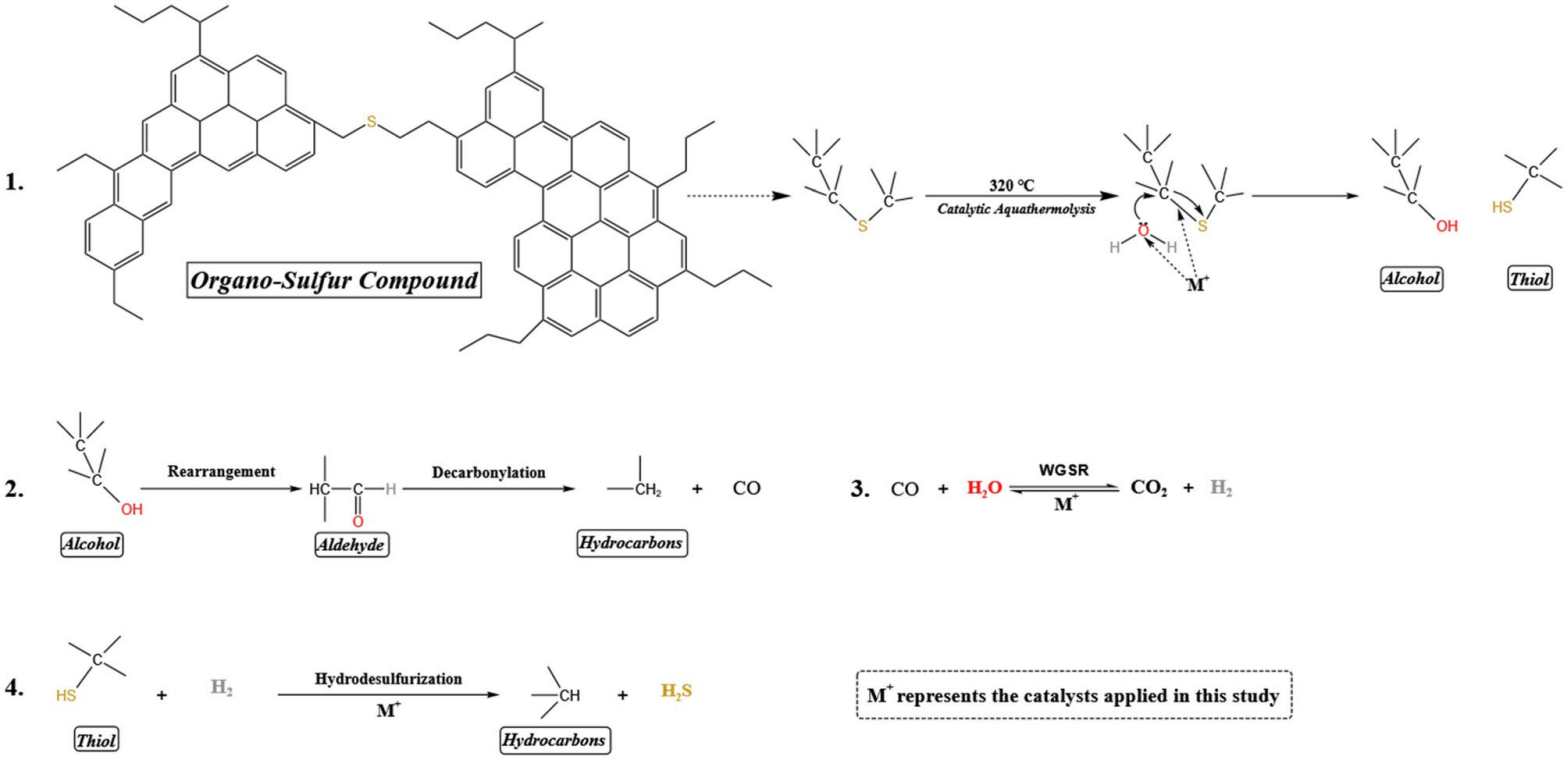
Reaction pathway for catalytic aquathermolysis by Hyne’s (1986) mechanism.

Based on the results discussed in the preceding sections, including sulfur content analysis, SARA analysis, viscosity measurements, and GC analysis of saturated hydrocarbons and evolved gases, it is observed that the cleavage of C–S bonds within asphaltene and resin structures, facilitated by the catalysts, promotes the conversion of large, complex molecules into smaller ones. This process results in a significant reduction in viscosity. This catalytic aquathermolysis process aligns with the mechanism proposed by Hyne (1986)^68^ for organo-sulfur compounds, as illustrated in Fig. 12. The mechanism involves several key steps: Step (1): Reaction with steam, where organo-sulfur compounds undergo hydrolysis in the presence of steam at 320 °C. This reaction, catalyzed by ZCFO and GO-ZCFO, leads to the formation of intermediate compounds, specifically alcohols (R-OH) and thiols (R-SH). Step (2): Alcohol rearrangement and aldehyde decarbonylation reaction, where alcohol molecules are rearranged into aldehydes. These aldehydes then undergo thermal degradation to produce hydrocarbons (R-H) and carbon monoxide (CO). Step (3): Water–gas shift reaction (WGSR), where the produced CO reacts with steam to form carbon dioxide (CO_2_) and hydrogen (H_2_). Step (4): Hydrodesulfurization reaction, where hydrogen generated via WGSR reacts with thiol compounds (R-SH) from Step (1) to form hydrocarbons (R-H) and hydrogen sulfide (H_2_S). Overall, the novel catalysts ZCFO and GO-ZCFO, with varying weight ratios, promote hydrolysis, WGSR, and hydrodesulfurization reactions, leading to a substantial reduction in sulfur content and viscosity, and thereby improving the overall quality of the upgraded heavy crude oil.

## Conclusion

One of the major challenges in upgrading heavy crude oil through catalytic aquathermolysis is achieving effective dispersion and stability of metal-based nanoparticles. Despite their high catalytic activity, these nanoparticles are prone to agglomeration, oxidation, and inadequate dispersion during synthesis and application. This study successfully addresses these limitations by employing graphene oxide (GO) as a carrier for metal nanoparticles, leading to the development of a novel GO-ZCFO nanocomposite. The incorporation of GO significantly enhances the stability and dispersion of the nanoparticles, thereby markedly improving their catalytic performance. In the upgrading process of heavy crude oil at 320 °C, comparative studies revealed the clear superiority and high efficiency of the GO-ZCFO composite over both steam alone and steam with ZCFO. The GO-ZCFO composite, particularly at a 50:50 weight ratio, demonstrated enhanced effectiveness in upgrading heavy crude oil by facilitating the conversion of higher molecular weight components into lighter fractions, as evidenced by the following key outcomes:

- A significant reduction in sulfur content from 5.117 to 3.62 wt.%.
- A notable decrease in resin content, from 17.77 to 13.99 wt.%.
- A marked reduction in asphaltene content, from 15.85 to 9.89 wt.%.
- A substantial increase in saturated hydrocarbon content, rising from 26.98 to 39.64 wt.%.
- A significant rise in low-molecular-weight hydrocarbons (C_8_–C_17_), coupled with a reduction in high-molecular-weight alkanes (C_18_–C_40_).
- An increase in the total amount of light hydrocarbon gases (n(C_1_–C_5_)).
- A considerable reduction in the viscosity of the upgraded oil, approximately threefold (from 1160.81 to 389.09 mPa^.^s).
- An observable increase in API gravity from 14.24° to 17.87°.

## Future research directions

- **Optimization of Operational Parameters:** Investigate the effects of varying temperatures, pressures, reaction times, and different catalyst concentrations on the upgrading process of heavy crude oil.
- **Post-Experiment Catalyst Characterization:** Investigate the morphological changes in the catalyst after the upgrading process to identify any structural modifications and assess their implications for catalyst stability and efficacy.
- **Core Flooding Experiments:** Conduct core flooding experiments to investigate the impact of viscosity reduction on heavy oil recovery enhancement.
- **Economic and Feasibility Analysis:** Perform an economic and feasibility analysis to evaluate the cost-effectiveness and practical viability of implementing GO-ZCFO nanocomposites in large-scale heavy oil upgrading operations.

## Supplementary information

Supplementary Information.

## Data Availability

All data generated or analysed during this study are included in this published article and its supplementary information files.

## References

[1] Zhang, Z., Barrufet, M., Lane, R. & Mamora, D. in *SPE Heavy Oil Conference Canada* .2012).

[2] Hein, F. J. Heavy oil and oil (tar) sands in north america: An overview & summary of contributions. *Nat. Resour. Res.* **15**, 67–84. 10.1007/s11053-006-9016-3 (2006).

[3] Selby, R., Alikhan, A. A. & Ali, S. M. F. Potential of non-thermal methods for heavy oil recovery. *J. Can. Pet. Technol.*10.2118/89-04-02 (1989).

[4] Mokrys, I. J. & Butler, R. M. in *SPE Production Operations Symposium.*

[5] Besson, C. *Resources to reserves: oil & gas technologies for the energy markets of the future*. (OECD/IEA, 2005).

[6] Guo, K., Li, H. & Yu, Z. In-situ heavy and extra-heavy oil recovery: A review. *Fuel* **185**, 886–902. 10.1016/j.fuel.2016.08.047 (2016).

[7] Zhao, F. *et al.* A review on upgrading and viscosity reduction of heavy oil and bitumen by underground catalytic cracking. *Energy Rep.* **7**, 4249–4272. 10.1016/j.egyr.2021.06.094 (2021).

[8] Mai, A., Bryan, J., Goodarzi, N. & Kantzas, A. Insights into non-thermal recovery of heavy oil. *J. Can. Pet. Technol.* **48**, 27–35. 10.2118/09-03-27 (2009).

[9] Miller, K. A. in *Canadian International Petroleum Conference* (2005).

[10] Alvarado, V. & Manrique, E. Enhanced oil recovery: An update review. *Energies* **3**, 1529–1575. 10.3390/en3091529 (2010).

[11] Nwidee, L. N., Theophilus, S., Barifcani, A., Sarmadivaleh, M. & Iglauer, S. *EOR processes, opportunities and technological advancements*. (InTech London, 2016).

[12] Shahat, J. S., Soliman, A. A., Gomaa, S. & Attia, A. M. Electrical tortuosity index: A new approach for identifying rock typing to enhance reservoir characterization using well-log data of uncored wells. *ACS Omega* **8**, 19509–19522. 10.1021/acsomega.3c00904 (2023). 37305282 10.1021/acsomega.3c00904 PMC10249038

[13] Soliman, A. A., El-Hoshoudy, A. N. & Attia, A. M. Assessment of xanthan gum and xanthan-g-silica derivatives as chemical flooding agents and rock wettability modifiers. *Oil Gas Sci. Technol.* **75**, 12. 10.2516/ogst/2020004 (2020).

[14] Aboelkhair, H., Diaz, P. & Attia, A. Biosurfactant production using Egyptian oil fields indigenous bacteria for microbial enhanced oil recovery. *J. Pet. Sci. Eng.* **208**, 109601. 10.1016/j.petrol.2021.109601 (2022).

[15] Sayyouh, M., Al-Blehed, M. & Attia, A. The effect of alkaline and polymer additives on phase behaviour of surfactant-oil-brine system at high salinity conditions. *Oil Gas Sci. Technol.* **48**, 359–369. 10.2516/ogst:1993023 (1993).

[16] Almalik, M. S., Attia, A. M. & Jang, L. K. Effects of alkaline flooding on the recovery of Safaniya crude oil of Saudi Arabia. *J. Pet. Sci. Eng.* **17**, 367–372. 10.1016/S0920-4105(96)00035-6 (1997).

[17] Azmi, G. E. *et al.* Adsorption of the xanthan gum polymer and sodium dodecylbenzenesulfonate surfactant in sandstone reservoirs: experimental and density function theory studies. *ACS Omega* **7**, 37237–37247. 10.1021/acsomega.2c03488 (2022). 36312333 10.1021/acsomega.2c03488 PMC9608398

[18] Pei, H., Zhang, G., Ge, J., Jin, L. & Ma, C. Potential of alkaline flooding to enhance heavy oil recovery through water-in-oil emulsification. *Fuel* **104**, 284–293. 10.1016/j.fuel.2012.08.024 (2013).

[19] Ali, S. M. F. & Thomas, S. The promise and problems of enhanced oil recovery methods. *J. Can. Pet. Technol.*10.2118/96-07-07 (1996).

[20] Li, P. *et al.* Synthesis and properties of the active polymer for enhanced heavy oil recovery. *Colloids Surf. A Physicochem. Eng. Asp.* **626**, 127036. 10.1016/j.colsurfa.2021.127036 (2021).

[21] Bryan, J. & Kantzas, A. in *SPE Annual Technical Conference and Exhibition* Vol. All Days (2007).

[22] Soliman, A. A., ElSahaa, M. A., Elsaeed, S. M., Zaki, E. G. & Attia, A. M. Sulfonamide derivatives as novel surfactant/alkaline flooding processes for improving oil recovery. *ACS Omega* **8**, 29401–29413. 10.1021/acsomega.3c02867 (2023). 37599960 10.1021/acsomega.3c02867 PMC10433505

[23] Elsaeed, S. M., Zaki, E. G., Omar, W. A. E., Ashraf Soliman, A. & Attia, A. M. Guar gum-based hydrogels as potent green polymers for enhanced oil recovery in high-salinity reservoirs. *ACS Omega* **6**, 23421–23431. 10.1021/acsomega.1c03352 (2021). 34549141 10.1021/acsomega.1c03352 PMC8444309

[24] Seyyedsar, S. M., Farzaneh, S. A. & Sohrabi, M. Experimental investigation of tertiary CO2 injection for enhanced heavy oil recovery. *J Nat Gas Sci Eng.* **34**, 1205–1214. 10.1016/j.jngse.2016.08.020 (2016).

[25] Huang, T., Zhou, X., Yang, H., Liao, G. & Zeng, F. CO2 flooding strategy to enhance heavy oil recovery. *Petroleum* **3**, 68–78. 10.1016/j.petlm.2016.11.005 (2017).

[26] Tan, Y. *et al.* A critical review of carbon dioxide enhanced oil recovery in carbonate reservoirs. *Fuel* **328**, 125256. 10.1016/j.fuel.2022.125256 (2022).

[27] Soliman, A. A., Gomaa, S., Shahat, J. S., El Salamony, F. A. & Attia, A. M. New models for estimating minimum miscibility pressure of pure and impure carbon dioxide using artificial intelligence techniques. *Fuel* **366**, 131374. 10.1016/j.fuel.2024.131374 (2024).

[28] Pratama, R. A. & Babadagli, T. A review of the mechanics of heavy-oil recovery by steam injection with chemical additives. *J. Pet. Sci. Eng.* **208**, 109717. 10.1016/j.petrol.2021.109717 (2022).

[29] Mahinpey, N., Ambalae, A. & Asghari, K. In situ combustion in enhanced oil recovery (EOR): A review. *Chem Eng Commun.* **194**, 995–1021. 10.1080/00986440701242808 (2007).

[30] Lashanizadegan, A., Ayatollahi, S. & Homayoni, M. Simultaneous heat and fluid flow in porous media: case study: steam injection for tertiary oil recovery. *Chem Eng Commun.* **195**, 521–535. 10.1080/00986440701709699 (2008).

[31] Speight, J. G. *Enhanced recovery methods for heavy oil and tar sands*. (Elsevier, 2009).

[32] Omole, O., Olieh, M. N. & Osinowo, T. Thermal visbreaking of heavy oil from the Nigerian tar sand. *Fuel* **78**, 1489–1496. 10.1016/S0016-2361(99)00023-X (1999).

[33] Hyne, J., Clark, P., Clarke, R., Koo, J. & Greidanus, J. Aquathermolysis of heavy oils. *J Rev. Tec. INTEVEP.* **2**, 87–94 (1982).

[34] Clark, P. D. & Hyne, J. B. Steam-oil chemical reactions: mechanisms for the aquathermolysis of heavy oils. *AOSTRA J. Res.* **1**, 15–20 (1984).

[35] Clark, P. D., Clarke, R. A., Hyne, J. B. & Lesage, K. L. Studies on the effect of metal species on oil sands undergoing steam treatments. *AOSTRA J. Res.* **6**, 53–64 (1990).

[36] Belgrave, J. D. M., Moore, R. G. & Ursenbach, M. G. Comprehensive kinetic models for the aquathermolysis of heavy oils. *J. Can. Pet. Technol.*10.2118/97-04-03 (1997).

[37] Maity, S. K., Ancheyta, J. & Marroquín, G. Catalytic aquathermolysis used for viscosity reduction of heavy crude oils: A review. *Energy Fuels.* **24**, 2809–2816. 10.1021/ef100230k (2010).

[38] Li, Y. *et al.* A review of in situ upgrading technology for heavy crude oil. *Petroleum* **7**, 117–122. 10.1016/j.petlm.2020.09.004 (2021).

[39] Kong, X. & Ohadi, M. M. in *Abu Dhabi International Petroleum Exhibition and Conference.*

[40] Rezk, M. Y. & Allam, N. K. Impact of nanotechnology on enhanced oil recovery: A mini-review. *Ind. Eng. Chem. Res.* **58**, 16287–16295. 10.1021/acs.iecr.9b03693 (2019).

[41] Hashemi, R., Nassar, N. N. & Pereira Almao, P. Nanoparticle technology for heavy oil in-situ upgrading and recovery enhancement: Opportunities and challenges. *Appl. Energy* **133**, 374–387. 10.1016/j.apenergy.2014.07.069 (2014).

[42] Hamedi Shokrlu, Y. & Babadagli, T. in *Canadian Unconventional Resources and International Petroleum Conference.*

[43] Hamedi Shokrlu, Y. & Babadagli, T. In-situ upgrading of heavy oil/bitumen during steam injection by use of metal nanoparticles: A study on in-situ catalysis and catalyst transportation. *SPE Reserv. Evaluation Eng.* **16**, 333–344. 10.2118/146661-PA (2013).

[44] Al-Marshed, A., Hart, A., Leeke, G., Greaves, M. & Wood, J. Optimization of heavy oil upgrading using dispersed nanoparticulate iron oxide as a catalyst. *Energy Fuels.* **29**, 6306–6316. 10.1021/acs.energyfuels.5b01451 (2015).

[45] Omajali, J. B., Hart, A., Walker, M., Wood, J. & Macaskie, L. E. In-situ catalytic upgrading of heavy oil using dispersed bionanoparticles supported on gram-positive and gram-negative bacteria. *Appl. Catal. B.* **203**, 807–819. 10.1016/j.apcatb.2016.10.074 (2017).

[46] Lam-Maldonado, M. *et al.* Transition metal nanocatalysts by modified inverse microemulsion for the heavy crude oil upgrading at reservoir. *Catalysis Today* **349**, 81–87. 10.1016/j.cattod.2018.05.052 (2020).

[47] Suwaid, M. A. *et al.* In-situ catalytic upgrading of heavy oil using oil-soluble transition metal-based catalysts. *Fuel* **281**, 118753. 10.1016/j.fuel.2020.118753 (2020).

[48] Al-Rubaye, A. H. *et al.* Intensification of the steam stimulation process using bimetallic oxide catalysts of MFe2O4 (M = Cu Co, Ni) for in-situ upgrading and recovery of heavy oil. *J. Pet. Explor. Prod. Technol.* **12**, 577–587. 10.1007/s13202-021-01311-1 (2022).

[49] Suwaid, M. A. *et al.* Using the oil-soluble copper-based catalysts with different organic ligands for in-situ catalytic upgrading of heavy oil. *Fuel* **312**, 122914. 10.1016/j.fuel.2021.122914 (2022).

[50] Wang, X. *et al.* Cis-9-Octadecenylamine modified ferric oxide and ferric hydroxide for catalytic viscosity reduction of heavy crude oil. *Fuel* **322**, 124159. 10.1016/j.fuel.2022.124159 (2022).

[51] Li, N., Ke, H., Wang, T. & Xia, S. Recyclable surface-functionalized Fe3O4 particles for heavy oil viscosity reduction. *J. Petrol. Sci. Eng.* **211**, 110112. 10.1016/j.petrol.2022.110112 (2022).

[52] Al-Muntaser, A. A. *et al.* Effect of decalin as hydrogen-donor for in-situ upgrading of heavy crude oil in presence of nickel-based catalyst. *Fuel* **313**, 122652. 10.1016/j.fuel.2021.122652 (2022).

[53] Mikhailova, A. N. *et al.* Ferrocene-based catalysts for in-situ hydrothermal upgrading of heavy crude oil: Synthesis and application. *Fuel* **348**, 128585. 10.1016/j.fuel.2023.128585 (2023).

[54] Liu, S., Wang, X., Zhou, H., Li, Q. & Yang, J. Oxalic acid as a hydrogen donor and its promotion on the catalytic viscosity reduction of heavy crude oil over Cu/FeOx catalyst. *Fuel* **357**, 130050. 10.1016/j.fuel.2023.130050 (2024).

[55] Sircar, A., Rayavarapu, K., Bist, N., Yadav, K. & Singh, S. Applications of nanoparticles in enhanced oil recovery. *Petrol. Res.* **7**, 77–90. 10.1016/j.ptlrs.2021.08.004 (2022).

[56] Khalil, M., Jan, B. M., Tong, C. W. & Berawi, M. A. Advanced nanomaterials in oil and gas industry: Design, application and challenges. *Appl. Energy* **191**, 287–310. 10.1016/j.apenergy.2017.01.074 (2017).

[57] Galukhin, A., Nosov, R., Eskin, A., Khelkhal, M. & Osin, Y. Manganese oxide nanoparticles immobilized on silica nanospheres as a highly efficient catalyst for heavy oil oxidation. *Ind. Eng. Chem. Res.* **58**, 8990–8995. 10.1021/acs.iecr.9b00080 (2019).

[58] Chi, M. *et al.* Silica Janus nanosheets anchored with Ni nanoparticles for in-situ upgrading and stimulus-responsive demulsification to enhance heavy oil recovery. *J. Anal. Appl. Pyrolysis* **181**, 106603. 10.1016/j.jaap.2024.106603 (2024).

[59] Wang, Y. *et al.* Experimental and mechanistic study on the effect of active components and calcination temperatures on biochar-based catalysts for catalyzing heavy oil viscosity reduction. *Geoenergy Sci. Eng.* **240**, 213078. 10.1016/j.geoen.2024.213078 (2024).

[60] Kumar, A., Rout, L., Achary, L. S. K., Dhaka, R. S. & Dash, P. Greener route for synthesis of aryl and alkyl-14H-dibenzo [a.j] xanthenes using graphene oxide-copper ferrite nanocomposite as a recyclable heterogeneous catalyst. *Sci. Rep.* **7**, 42975. 10.1038/srep42975 (2017). 28233832 10.1038/srep42975 PMC5324042

[61] Baig, R. B. N. & Varma, R. S. Magnetically retrievable catalysts for organic synthesis. *Chem. Commun.* **49**, 752–770 (2013). 10.1039/c2cc35663e 23212208

[62] Shylesh, S., Schünemann, V. & Thiel, W. R. Magnetically separable nanocatalysts: Bridges between homogeneous and heterogeneous catalysis. *Angew Chem. Int. Ed. Engl.* **49**, 3428–3459. 10.1002/anie.200905684 (2010). 20419718 10.1002/anie.200905684

[63] Zhu, X. *et al.* Control of metal-support interaction in magnetic MoS2 catalyst to enhance hydrodesulfurization performance. *J. Environ. Chem. Eng.* **9**, 106109. 10.1016/j.jece.2021.106109 (2021).

[64] Rossi, L. M., Costa, N. J. S., Silva, F. P. & Wojcieszak, R. Magnetic nanomaterials in catalysis: advanced catalysts for magnetic separation and beyond. *Green Chem.* **16**, 2906–2933 (2014).

[65] Marcano, D. C. *et al.* Improved synthesis of graphene oxide. *ACS Nano* **4**, 4806–4814. 10.1021/nn1006368 (2010). 20731455 10.1021/nn1006368

[66] Huang, Y. *et al.* Degradation of atrazine by ZnxCu1−xFe2O4 nanomaterial-catalyzed sulfite under UV–vis light irradiation: Green strategy to generate SO4−. *Appl. Catal. B.* **221**, 380–392. 10.1016/j.apcatb.2017.09.001 (2018).

[67] Yu, R., Zhao, J., Zhao, Z. & Cui, F. Copper substituted zinc ferrite with abundant oxygen vacancies for enhanced ciprofloxacin degradation via peroxymonosulfate activation. *J. Hazard. Mater.* **390**, 121998. 10.1016/j.jhazmat.2019.121998 (2020). 32044618 10.1016/j.jhazmat.2019.121998

[68] Hyne, J. B. *Aquathermolysis: A Synopsis of Work on the Chemical Reaction Between Water (steam) and Heavy Oil Sands During Simulated Steam Stimulation*. (Alberta Oil Sands Technology and Research Authority, 1986).

[69] Shakirullah, M., Ahmad, I., Ishaq, M. & Ahmad, W. Catalytic hydro desulphurization study of heavy petroleum residue through in situ generated hydrogen. *Energy Convers. Manage.* **51**, 998–1003. 10.1016/j.enconman.2009.12.002 (2010).

[70] Alboudwarej, H., Beck, J., Svrcek, W. Y., Yarranton, H. W. & Akbarzadeh, K. Sensitivity of Asphaltene Properties to Separation Techniques. *Energy Fuels* **16**, 462–469. 10.1021/ef010213p (2002).

[71] Astm, D. *4124, Standard Test Method for separation of asphalt into four fractions* (American Society for Testing and Material, 1988).

[72] Tony Dhiwahar, A., Sundararajan, M., Sakthivel, P., Dash, C. S. & Yuvaraj, S. Microwave-assisted combustion synthesis of pure and zinc-doped copper ferrite nanoparticles: Structural, morphological, optical, vibrational, and magnetic behavior. *J. Phys. Chem. Solids.* **138**, 109257. 10.1016/j.jpcs.2019.109257 (2020).

[73] Givalou, L. *et al.* Transition metal – Graphene oxide nanohybrid materials as counter electrodes for high efficiency quantum dot solar cells. *Catal. Today.* **355**, 860–869. 10.1016/j.cattod.2019.03.035 (2020).

[74] Li, D., Müller, M. B., Gilje, S., Kaner, R. B. & Wallace, G. G. Processable aqueous dispersions of graphene nanosheets. *Nat. Nanotechnol.* **3**, 101–105. 10.1038/nnano.2007.451 (2008). 18654470 10.1038/nnano.2007.451

[75] Meidanchi, A. & Akhavan, O. Superparamagnetic zinc ferrite spinel–graphene nanostructures for fast wastewater purification. *Carbon* **69**, 230–238. 10.1016/j.carbon.2013.12.019 (2014).

[76] Matloubi Moghaddam, F., Doulabi, M. & Saeidian, H. Controlled microwave-assisted synthesis of ZnFe2O4 nanoparticles and their catalytic activity for O-acylation of alcohol and phenol in acetic anhydride. *Sci. Iran.* **19**, 1597–1600. 10.1016/j.scient.2012.10.013 (2012).

[77] Nawle, A. C., Humbe, A. V., Babrekar, M. K., Deshmukh, S. S. & Jadhav, K. M. Deposition, characterization, magnetic and optical properties of Zn doped CuFe2O4 thin films. *J. Alloys Compd.* **695**, 1573–1582. 10.1016/j.jallcom.2016.10.301 (2017).

[78] Ahmed, M. A., Ahmed, M. A. & Mohamed, A. A. Facile adsorptive removal of dyes and heavy metals from wastewaters using magnetic nanocomposite of zinc ferrite@reduced graphene oxide. *Inorg. Chem. Commun.* **144**, 109912. 10.1016/j.inoche.2022.109912 (2022).

[79] Cheshkova, T. V. *et al.* Resins and asphaltenes of light and heavy oils: Their composition and structure. *Energy Fuels.* **33**, 7971–7982. 10.1021/acs.energyfuels.9b00285 (2019).

[80] Esmaeilian, N., Rabiei, N., Mahmoudi, M. & Dabir, B. Asphaltene structure determination: FTIR, NMR, EA, ICP-OES, MS, XRD and computational chemistry considerations. *J. Mol. Liq.* **385**, 122279. 10.1016/j.molliq.2023.122279 (2023).

[81] Demirbaş, A. Removing of resins from crude oils. *Pet Sci Technol.* **34**, 771–777. 10.1080/10916466.2016.1163397 (2016).

[82] Dharaskar, S. A. *et al.* Synthesis, characterization, and application of novel trihexyl tetradecyl phosphonium bis (2,4,4-trimethylpentyl) phosphinate for extractive desulfurization of liquid fuel. *Fuel Process. Technol.* **123**, 1–10. 10.1016/j.fuproc.2014.02.001 (2014).

[83] Tang, X. D., Chen, X. D., Li, J. J., Deng, L. Y. & Liang, G. J. Experimental study on homogeneous catalytic upgrading of heavy oil. *Pet. Chem.* **57**, 1018–1023. 10.1134/S0965544117120143 (2017).

[84] Vakhin, A. V. *et al.* Extra-heavy oil aquathermolysis using nickel-based catalyst: Some aspects of in-situ transformation of catalyst precursor. *Catalysts* **11**, 189. 10.3390/catal11020189 (2021).

[85] Gunugunuri, K. & Smirniotis, p. *Water Gas Shift Reaction: Research Developments and Applications*. (Elsevier, 2015).

