# Supplementary material for: Optimizing in-situ upgrading of heavy crude oil via catalytic aquathermolysis using a novel graphene oxide-copper zinc ferrite nanocomposite as a catalyst
Source: Sci Rep. 2024 Oct 28;14:25845. doi: 10.1038/s41598-024-73953-0 (PMC11519547; doi:10.1038/s41598-024-73953-0)
Supplement: Supplementary file 1 — Supplementary Information. [file 41598_2024_73953_MOESM1_ESM.docx]

**Supplementary Material for Optimizing In-Situ Upgrading of Heavy Crude Oil via Catalytic Aquathermolysis using a Novel Graphene Oxide-Copper Zinc Ferrite Nanocomposite as a Catalyst**

**Ahmed Ashraf Soliman^1,2,5,*^, Mostafa E. Aboul-Fetouh^1^, Sayed Gomaa^1,3^, Tarek M. Aboul-Fotouh^1,3,4^, Attia Mahmoud Attia^5^**

^1^Mining and Petroleum Engineering Department, Faculty of Engineering, Al-Azhar University, Nasr City, Cairo 11884, Egypt.

^2^Khalda Petroleum Company, Ministry of Petroleum, Egypt.

^3^Petroleum Engineering Department, Faculty of Engineering and Technology, Future University in Egypt, Cairo 11835, Egypt.

^4^Chemical Engineering Department, Faculty of Engineering, British University in Egypt (BUE), El Sherouk City, Cairo, Egypt.

^5^Petroleum Engineering and Gas Technology Department, Faculty of Energy and Environmental Engineering, British University in Egypt (BUE), El Sherouk City, Cairo, Egypt.

**Contents of This File**

- Introduction
- Table S-1
- References

**Introduction**

Table S-1 illustrates the distribution of carbon numbers and the content of saturated hydrocarbons before and after upgrading processes for heavy crude oil. Non-catalytic aquathermolysis exhibited minimal changes in low-molecular-weight alkane content. However, the introduction of catalysts led to a significant increase in these lighter hydrocarbons and a simultaneous decrease in heavier alkanes. These findings indicate that catalysts play a crucial role in breaking down heavy hydrocarbon chains during aquathermolysis, resulting in the formation of lighter fractions and consequently reducing the viscosity of heavy crude oil.

Table S-1. The carbon number distribution and content in the saturated hydrocarbons before and after upgrading at 320°C.

|  | **Original crude Oil** | **Crude oil with steam** | **Crude oil with steam and catalyst**  **based on** | | | |
| --- | --- | --- | --- | --- | --- | --- |
|  |  |  | **ZCFO** | **GO-ZCFO (30:70)** | **GO-ZCFO (50:50)** | **GO-ZCFO (70:30)** |
| **C_8_** | 2.668 | 4.715 | 3.491 | 3.428 | 5.048 | 4.383 |
| **C_9_** | 8.31 | 5.913 | 4.362 | 7.215 | 3.907 | 3.303 |
| **C_10_** | 7.948 | 7.391 | 8.875 | 9.895 | 6.911 | 8.893 |
| **C_11_** | 7.667 | 8.353 | 6.312 | 10.204 | 8.637 | 10.227 |
| **C_12_** | 7.151 | 9.625 | 12.287 | 10.471 | 10.382 | 8.399 |
| **C_13_** | 9.672 | 10.937 | 10.348 | 11.185 | 15.684 | 9.047 |
| **C_14_** | 5.904 | 7.412 | 7.128 | 11.883 | 12.316 | 14.471 |
| **C_15_** | 5.906 | 6.186 | 7.559 | 7.886 | 8.983 | 8.901 |
| **C_16_** | 4.557 | 5.242 | 6.561 | 6.686 | 7.15 | 5.436 |
| **C_17_** | 4.533 | 3.736 | 7.187 | 5.948 | 6.871 | 6.518 |
| **C_18_** | 3.453 | 2.357 | 5.678 | 4.981 | 5.854 | 5.857 |
| **C_19_** | 4.521 | 3.45 | 4.196 | 2.56 | 2.689 | 4.375 |
| **C_20_** | 3.16 | 3.055 | 3.572 | 1.718 | 1.871 | 3.378 |
| **C_21_** | 4.846 | 3.35 | 2.857 | 1.51 | 1.161 | 2.12 |
| **C_22_** | 2.543 | 3.405 | 2.541 | 1.176 | 0.877 | 1.701 |
| **C_23_** | 2.349 | 2.091 | 1.504 | 1.05 | 0.595 | 0.649 |
| **C_24_** | 2.086 | 2.052 | 1.203 | 0.711 | 0.338 | 0.625 |
| **C_25_** | 2.003 | 1.915 | 1.071 | 0.455 | 0.283 | 0.424 |
| **C_26_** | 1.872 | 1.576 | 0.869 | 0.307 | 0.132 | 0.405 |
| **C_27_** | 1.764 | 1.468 | 0.847 | 0.208 | 0.095 | 0.252 |
| **C_28_** | 1.747 | 1.432 | 0.501 | 0.194 | 0.082 | 0.202 |
| **C_29_** | 1.639 | 1.382 | 0.423 | 0.098 | 0.052 | 0.108 |
| **C_30_** | 1.606 | 1.022 | 0.257 | 0.078 | 0.038 | 0.085 |
| **C_31_** | 0.521 | 0.628 | 0.115 | 0.05 | 0.028 | 0.067 |
| **C_32_** | 0.421 | 0.413 | 0.085 | 0.037 | 0.016 | 0.053 |
| **C_33_** | 0.312 | 0.364 | 0.074 | 0.028 |  | 0.049 |
| **C_34_** | 0.271 | 0.251 | 0.058 | 0.027 |  | 0.04 |
| **C_35_** | 0.175 | 0.138 | 0.039 | 0.011 |  | 0.032 |
| **C_36_** | 0.126 | 0.084 |  |  |  |  |
| **C_37_** | 0.101 | 0.039 |  |  |  |  |
| **C_38_** | 0.082 | 0.018 |  |  |  |  |
| **C_39_** | 0.042 |  |  |  |  |  |
| **C_40_** | 0.044 |  |  |  |  |  |
